# Identifying mutation hotspots reveals pathogenetic mechanisms of *KCNQ2* epileptic encephalopathy

**DOI:** 10.1038/s41598-020-61697-6

**Published:** 2020-03-16

**Authors:** Jiaren Zhang, Eung Chang Kim, Congcong Chen, Erik Procko, Shashank Pant, Kin Lam, Jaimin Patel, Rebecca Choi, Mary Hong, Dhruv Joshi, Eric Bolton, Emad Tajkhorshid, Hee Jung Chung

**Affiliations:** 1 Department of Molecular and Integrative Physiology, University of Illinois at Urbana-Champaign, Urbana, Illinois 61801 USA; 2 Department of Statistics, University of Illinois at Urbana-Champaign, Urbana, Illinois 61801 USA; 3 Department of Biochemistry, University of Illinois at Urbana-Champaign, Urbana, Illinois 61801 USA; 4 Neuroscience Program, University of Illinois at Urbana-Champaign, Urbana, Illinois 61801 USA; 5 NIH Center for Macromolecular Modeling and Bioinformatics, Beckman Institute for Advanced Science and Technology, University of Illinois at Urbana-Champaign, Urbana, Illinois 61801 USA; 6 Center for Biophysics and Quantitative Biology, University of Illinois at Urbana-Champaign, Urbana, Illinois 61801 USA; 7 Department of Physics, University of Illinois at Urbana-Champaign, Urbana, Illinois 61801 USA

**Keywords:** Ion channels in the nervous system, Neurophysiology

## Abstract

K_v_7 channels are enriched at the axonal plasma membrane where their voltage-dependent potassium currents suppress neuronal excitability. Mutations in K_v_7.2 and K_v_7.3 subunits cause epileptic encephalopathy (EE), yet the underlying pathogenetic mechanism is unclear. Here, we used novel statistical algorithms and structural modeling to identify EE mutation hotspots in key functional domains of K_v_7.2 including voltage sensing S4, the pore loop and S6 in the pore domain, and intracellular calmodulin-binding helix B and helix B-C linker. Characterization of selected EE mutations from these hotspots revealed that L203P at S4 induces a large depolarizing shift in voltage dependence of K_v_7.2 channels and L268F at the pore decreases their current densities. While L268F severely reduces expression of heteromeric channels in hippocampal neurons without affecting internalization, K552T and R553L mutations at distal helix B decrease calmodulin-binding and axonal enrichment. Importantly, L268F, K552T, and R553L mutations disrupt current potentiation by increasing phosphatidylinositol 4,5-bisphosphate (PIP_2_), and our molecular dynamics simulation suggests PIP_2_ interaction with these residues. Together, these findings demonstrate that each EE variant causes a unique combination of defects in K_v_7 channel function and neuronal expression, and suggest a critical need for both prediction algorithms and experimental interrogations to understand pathophysiology of K_v_7-associated EE.

## Introduction

Epilepsy is the second most prominent neurological disease (www.epilepsy.com), in which excessive electrical activity within networks of neurons in the brain manifests clinically as recurrent unprovoked seizures^1^. Recent discoveries of epilepsy-related genes in multiple laboratories and through large consortia have revealed a diverse array of proteins that may contribute to epileptogenesis^1,2^. Among these proteins, neuronal KCNQ/K_v_7 potassium (K^+^) channels have been implicated in epilepsy since mutations in the principle subunits, KCNQ2/K_v_7.2 and KCNQ3/K_v_7.3, cause Benign Familial Neonatal Epilepsy (BFNE [MIM: 121200]) and Epileptic Encephalopathy (EE [MIM: 613720]) (RIKEE database www.rikee.org).

Neuronal K_v_7 channels are mainly composed of heterotetramers of K_v_7.2 and K_v_7.3^3^, which show overlapping distribution in the hippocampus and cortex^4^. They generate slowly activating and non-inactivating voltage-dependent K^+^ currents that contribute to resting membrane potential, prevent repetitive and burst firing of action potentials (APs), and modulate AP threshold^3,5–7^.They are enriched at the plasma membrane of axonal initial segments (AIS) and distal axons^8,9^, where APs initiate and propagate^10^. Membrane phosphatidylinositol-4,5-bisphosphate (PIP_2_) is required for K_v_7 channels to open^3^, although its exact binding sites in K_v_7.2 and K_v_7.3 are still under investigation^11–15^. They are also called ‘M-channels’ because their currents are inhibited by PIP_2_ depletion upon activation of Gq/11-coupled receptors such as the M1 muscarinic acetylcholine receptor^14,16,17^.

Nearly 200 BFNE and EE mutations in *KCNQ2* and *KCNQ3* genes have been identified to date (RIKEE database www.rikee.org). Dominantly inherited BFNE variants cause neonatal seizures that show spontaneous remission with benign psychomotor and intellectual outcomes^18^, and their effects on K_v_7 channel function and excitability have been extensively studied^19,20^. A large number of *de novo* EE variants in *KCNQ2* have been recently discovered since 2012^2,21–26^. Patients with EE variants display early-onset seizures, developmental delay, neuroradiological abnormalities, and behavioral comorbidities including intellectual disability and autism^21,23–25^. Current anti-epileptic drugs are ineffective in treating many patients with *KCNQ2* EE variants^21,25,27^, posing a critical need to understand how EE mutations disrupt K_v_7 channels and lead to severe symptomatic epilepsy.

One important step is to determine if EE variants cluster at key functional domains that are critical for voltage-dependent gating and expression of K_v_7 channels. Each K_v_7 subunit contains 6 transmembrane segments (S1–S6)^3,28^. The S1-S4 segments form voltage sensing domains with S4 as a main sensor for depolarization^3,28,29^. The pore loop between S5 and S6 contains a highly conserved selectivity filter that controls K^+^ permeability and selectivity^3,28,29^. The C-terminal intersection of four S6 segments constitutes the main gate^28,29^. The intracellular C-terminal tails of K_v_7.2 and K_v_7.3 contain sites for PIP_2_-dependent modulation and ankyrin-G-dependent targeting to the AIS, and 4 helical structures (helices A-D) that mediate channel assembly through helix C and interaction with calmodulin (CaM) through helices A and B^8,30^. Therefore, we hypothesize that EE mutations are enriched at specific functional domains of K_v_7.2 and disrupt their functions.

In this study, we test this hypothesis by developing novel statistical algorithms and modeling of K_v_7.2, since computational *in-silico* algorithms have been shown to be useful tools for predicting pathogenicity of sequence variants^31^. We show that EE variants are significantly clustered at S4, the pore loop, S6, helix B, and the helix B-C linker of K_v_7.2. Our investigation of selected EE variants in epilepsy mutation hotspots revealed that each mutation impaired the function of its associated protein domain. Unexpectedly, we discovered that selected EE mutations reside in the PIP_2_ binding regions of K_v_7.2. Furthermore, selected mutations in the pore loop and helix B impaired K_v_7 current enhancement upon increasing PIP_2_ and severely decreased surface expression of heteromeric channels in neurons. These findings emphasize the importance of both prediction algorithms and experimental interrogations to understand pathophysiology of K_v_7-associated EE.

## Results

### MHF algorithm identifies EE mutation clusters in K_v_7.2

**Figure 1:**
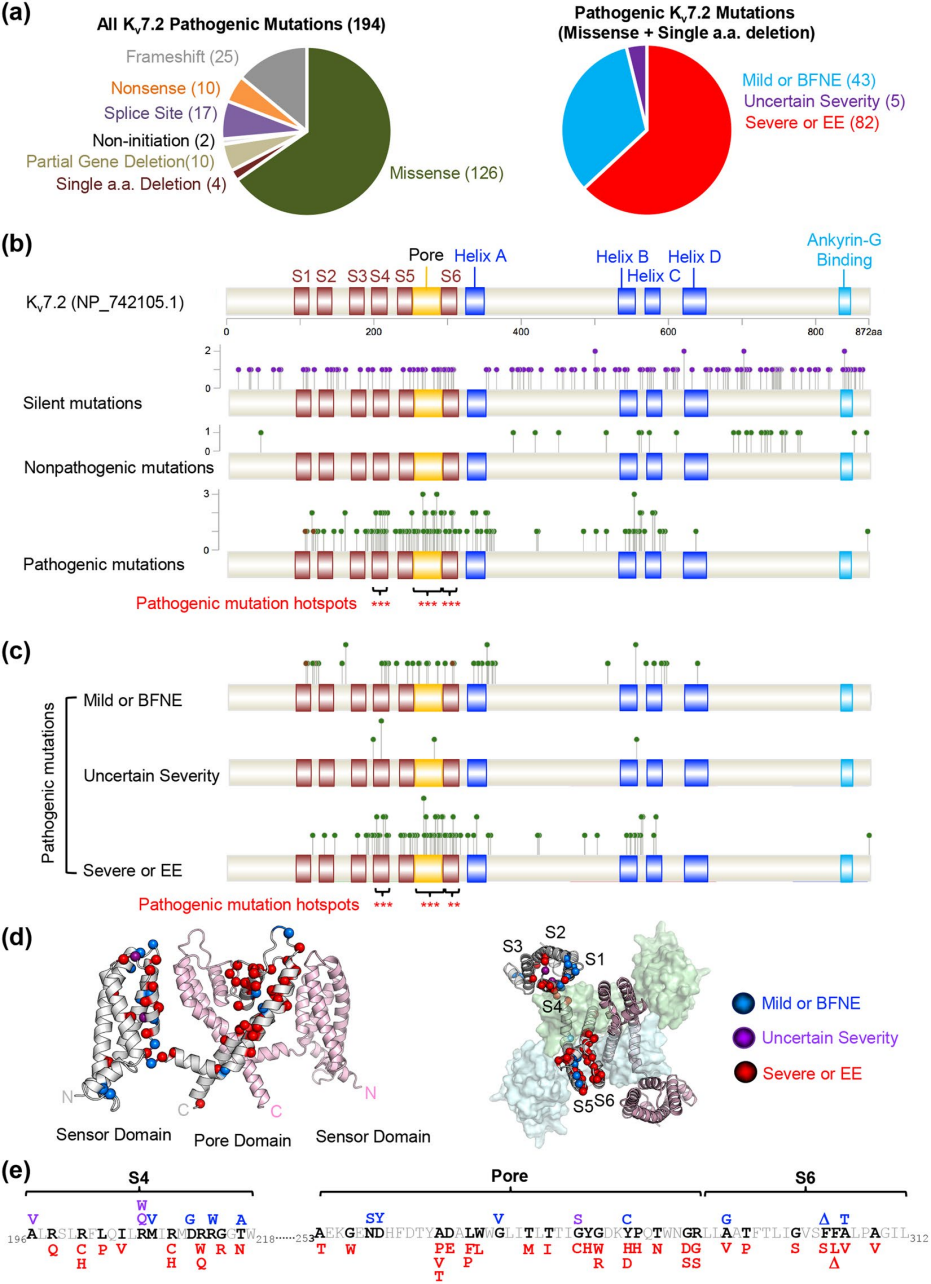
Epilepsy mutations cluster at the S4, the pore, and the S6 domains of K_v_7.2. (**a**) Pie charts showing the relative proportions of different types of epilepsy variants in human K_v_7.2 (left) and of single amino acid K_v_7.2 mutations that cause epilepsy with different clinical severity including “mild or BFNE”, “uncertain severity”, and “severe or epileptic encephalopathy (EE)” (right). All mutations identified up to December 31, 2017 are shown in Supplementary Table S1. (**b**) Single amino acid mutations except for 2 missense mutations in the primary start ATG codon (green markers) and silent mutations (purple markers) are mapped to K_v_7.2 primary structure (NP_742105.1). The MHF statistical algorithm identified S4, the pore loop, and S6 as hotspots for all pathogenic epilepsy mutations (brackets, ***p < 0.005, Supplementary Table S2). (**c**) The MHF algorithm revealed S4, the pore loop, and S6 as hotspots for “severe or EE” mutations (brackets, **p < 0.01, ***p < 0.005, Supplementary Table S3). (**d**) The S1-S6 domains of tetrameric human K_v_7.2 were modeled based on the cryoEM structure of Xenopus K_v_7.1 (Protein Data Bank: 5VMS)^28^. At left, two opposing subunits are viewed from the plane of the plasma membrane. At right, the model is viewed from the extracellular space, with two opposing subunits shown as ribbons and their neighbors shown as transparent surfaces. Sites of pathogenic mutations are highlighted with colored spheres on the C-alpha atoms of one subunit: mild or BFNE (blue), uncertain severity (purple), severe or EE (red). Where more than one mutation occurs at a single position, the residue is colored by the most severe phenotype. (**e**) Location of amino acids mutated in mild or BFNE (blue), uncertain severity (purple), or severe or EE (red) in S4, the pore loop and S6 of K_v_7.2.

We compiled genetic information and clinical symptoms of 194 epilepsy mutations in K_v_7.2 that were reported until December 31, 2017 (Fig. 1a, Supplementary Table S1). These variants include 10 submicroscopic and partial gene deletions, 17 splice site mutations, 10 nonsense mutations, 25 frameshift mutations, 2 non-initiation mutations, 126 missense mutations that lead to single amino acid substitutions, and 4 mutations that result in single amino acid deletions (Fig. 1a). These mutations were classified into three groups according to the severities of their clinical outcomes described in the RIKEE database (Fig. 1a, Supplementary Table S1). The “mild or BFNE” mutations lead to seizures but not developmental delay in patients. The “severe or EE” mutations cause neonatal encephalopathy, seizures, and developmental delays. The “uncertain severity” mutations are associated with both benign seizures and EE or have limited clinical information. In addition, 130 silent mutations (with highest allele frequency from 0.0017% to 19%) and 25 relatively common nonpathogenic missense mutations of K_v_7.2 (with allele frequency ≥ 0.01%) were identified from the Exome Aggregation Consortium (ExAC) database that collected protein-coding genetic variations from 60,706 humans (http://exac.broadinstitute.org).

In contrast to the evenly distributed silent mutations, pathogenic single amino acid mutations are concentrated at transmembrane segments S1 to S6, the pore loop, and intracellular helices A and B of K_v_7.2 (Fig. 1b). To test if this trend was statistically significant, we developed a resampling algorithm titled Mutation Hotspot Finder (MHF). This algorithm was applied under the null hypothesis that pathogenic mutations are equally observed at every residue of a functional domain in the full-length K_v_7.2 protein when there is no further association between the mutations and the domains. Because our MHF examines the association between the pathogenic variants and the functional domains, we used 130 single amino acid mutations and excluded nonsense and frameshift mutations that truncate one or more functional domains in K_v_7.2. This analysis revealed that epilepsy mutations are significantly clustered at the voltage sensing S4, the pore loop, and S6 of K_v_7.2 (p < 0.005), whereas silent and nonpathogenic mutations did not cluster at any of the functional domains (Fig. 1b, Supplementary Table S2). Importantly, epilepsy mutations of K_v_7.2 were significantly associated with the “severe or EE” group (*p* < 0.001) and not the “mild or BFNE” and “uncertain severity” groups (Fig. 1a–e, Supplementary Table S3).

**Figure 2:**
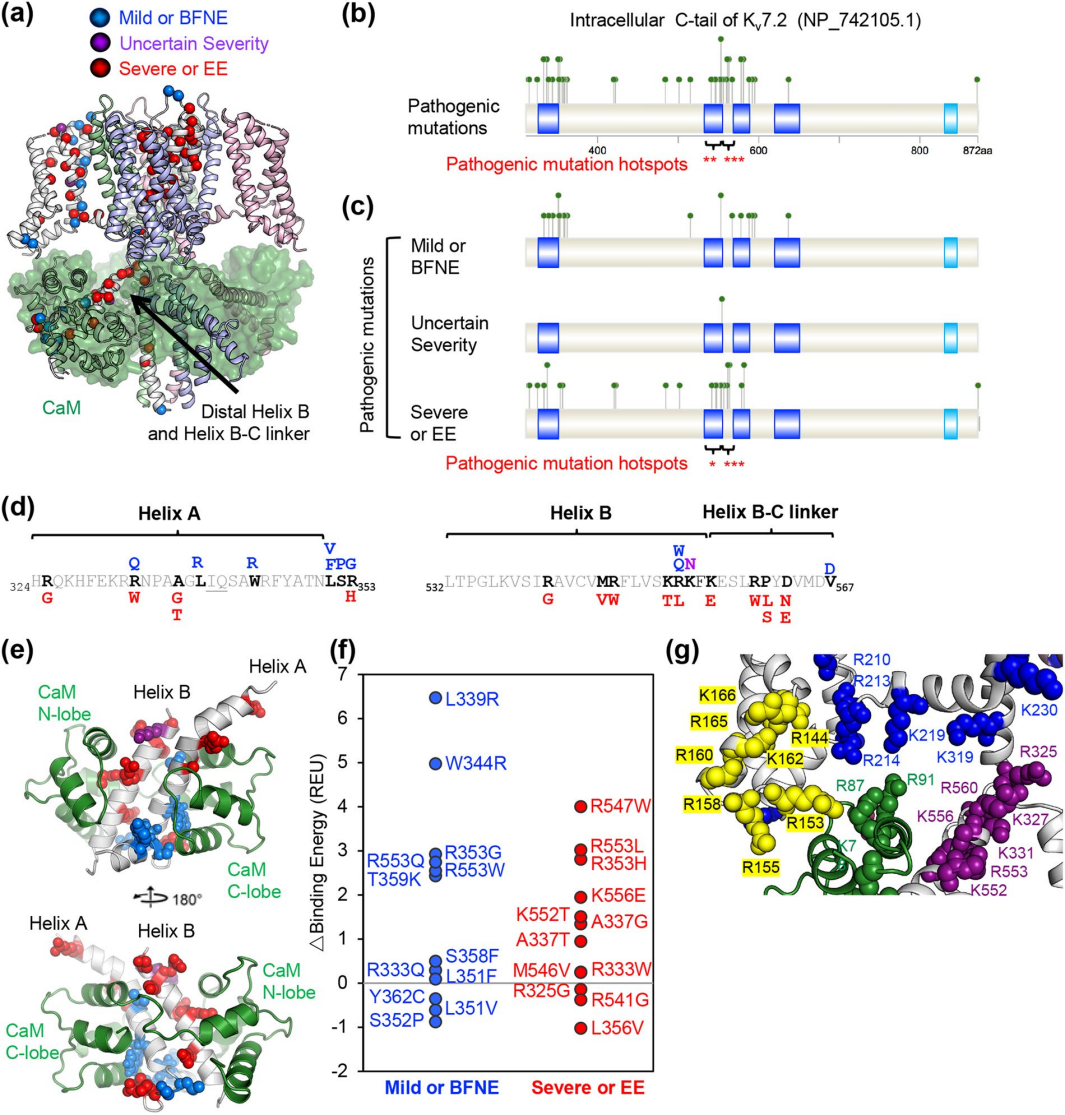
Epilepsy mutations cluster at CaM-binding helix B and the helix B-C linker of K_v_7.2. (**a**) Tetrameric human K_v_7.2 (ribbons) in complex with four CaM subunits (transparent green surfaces) was modeled based on the structure of Xenopus K_v_7.1 (Protein Data Bank: 5VMS)^28^. Sites of pathogenic mutations in K_v_7.2 C-terminal tail are highlighted with colored spheres on the C-alpha atoms of one subunit: mild or BFNE (blue), uncertain severity (purple), severe or EE (red). In addition to epilepsy mutation hotspots (S4, the pore loop, and S6), “severe or EE” mutations cluster near the inner leaflet of the plasma membrane and the C-terminus of S6 at the base of the gate. (**b**,**c**) The MHF statistical algorithm on the intracellular C-terminal tail of K_v_7.2 identified helix B and the helix B-C linker as hotspots for all pathogenic epilepsy mutations (brackets, **p < 0.01, ***p < 0.005, Supplementary Table S2) (**b**) and EE mutations (brackets, *p < 0.05, ***p < 0.005, Supplementary Table S3) (**c**). (**d**) Location of amino acids mutated in mild or BFNE (blue), uncertain severity (purple), or severe or EE (red) in helix A containing a consensus IQ motif for binding CaM (underlined), helix B, and the helix B-C linker of K_v_7.2. (**e**) Mutated amino acids are highlighted on a model of K_v_7.2 helix A and B (grey) bound to Ca^2+^-CaM (green), which was modeled after the crystal structure of chimeric K_v_7.3 helix A - K_v_7.2 helix B protein in complex with Ca^2+^-CaM (Protein Data Bank: 5J03)^32^. Side chains for residues with pathogenic mutations are colored spheres: mild or BFNE (blue), uncertain severity (purple), severe or EE (red). (**f**) Predicted changes in Ca^2+^-CaM binding energy of pathogenic K_v_7.2 missense mutations within helices A and B. The higher the energy the weaker the predicted affinity for Ca^2+^-CaM. (**g**) Positively charged basic residues at proximal helix A, distal helix B and the helix B-C linker (purple) are located close to basic residues from the S2–3 linker (yellow), S4, S6 (blue), and CaM (green).

Our MHF analysis also revealed that helix B and helix B-C linker have significantly more pathogenic mutations (p < 0.01) than other domains within the K_v_7.2 C-terminal tail due to the clustering of “severe or EE” mutations (*p* < 0.05)(Fig. 2a–d, Supplementary Tables S2–3. Since K_v_7.2 binds to CaM through helices A and B (Fig. 2e)^28,32^, we next tested if the clinical severity of epilepsy mutations is associated with the extent to which K_v_7.2 variants bind to CaM. Both “mild or BFNE” and “severe or EE” mutations located at helices A and B decreased the CaM binding energy of K_v_7.2 (Fig. 2e,f). Furthermore, EE mutations occur at the positively charged residues in the distal portion of helix B and the helix B-C linker away from the CaM contact site in the modeled K_v_7.2 structure (Fig. 2a,g). These results suggest that disruption of CaM binding alone cannot explain why “severe or EE” mutations were selectively enriched at helix B and the helix B-C linker.

### Voltage-dependent activation of homomeric K_v_7.2 channel is disrupted by selected EE mutations in epilepsy mutation hotspots

**Figure 3:**
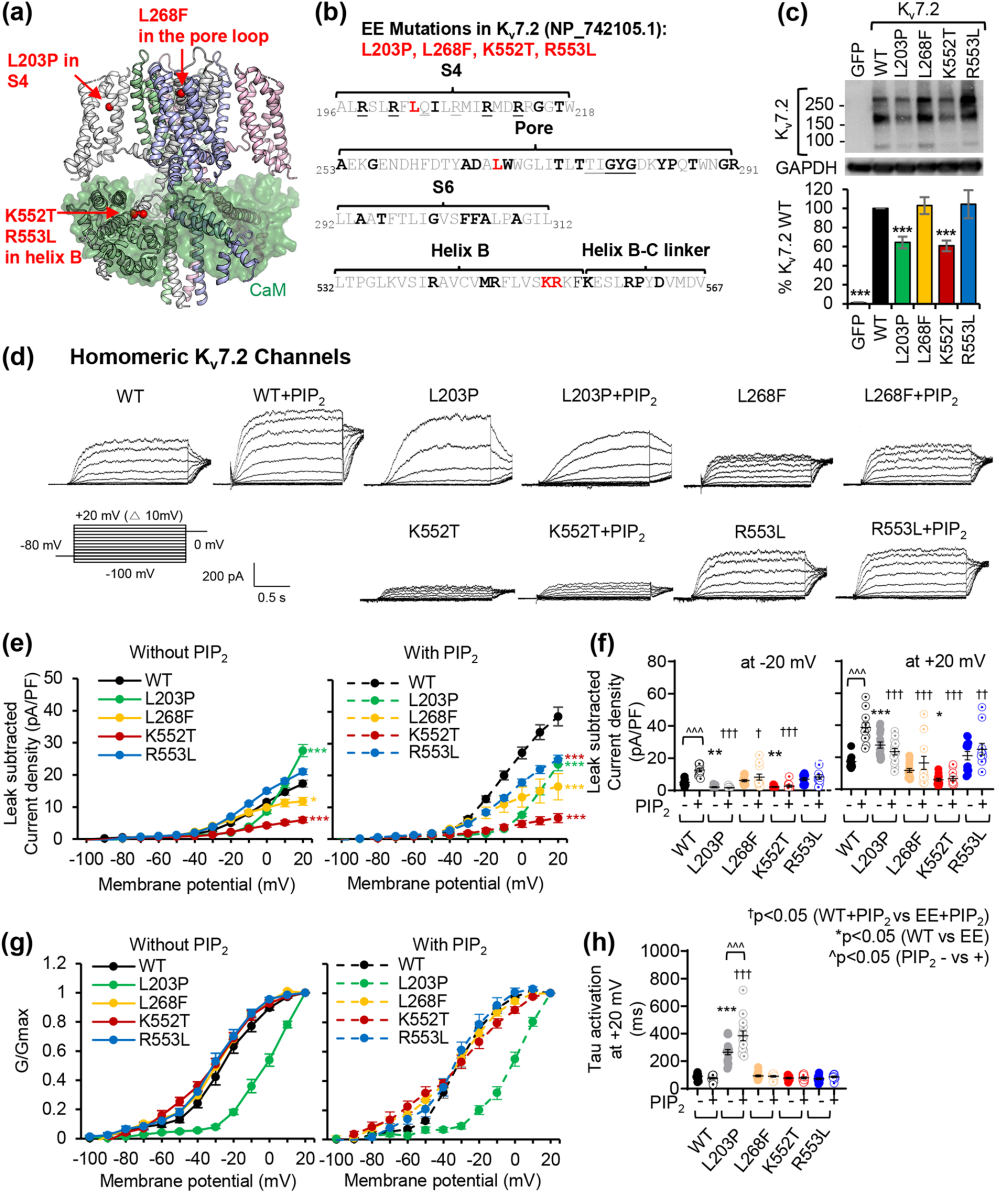
All selected EE mutations variably alter voltage-dependent activation of homomeric K_v_7.2 channels and disrupt their current enhancement upon diC8-PIP_2_ inclusion. (**a**) Sites of selected EE mutations (L203P, L268F, K552T, and R553L) characterized in this study. These mutations are highlighted with red spheres on the C-alpha atoms of one subunit on the modeled tetrameric human K_v_7.2 structure (ribbons) in complex with four CaM subunits (transparent green surfaces). (**b**) Localization of selected EE mutations are shown in red in the amino acid sequence of K_v_7.2 (NP_742105.1). The EE mutations are shown in bold. The critical residues in S4 and the selectivity filter in the pore are underlined. (**c–h**) Whole cell voltage clamp recordings of macroscopic K^+^ currents in CHO hm1 cells transfected with GFP and K_v_7.2 WT or EE mutants. Cells were held at -80 mV. Currents were evoked by depolarization for 1.5 s from −100 mV to +20 mV in 10 mV increments, followed by a step to 0 mV for 300 ms. To examine PIP_2_ sensitivity of K_v_7.2 channels, the recording was repeated with internal patch pipette solution containing diC8-PIP_2_ (100 μM) which also contained EGTA to sequester free Ca^2+^. The raw current traces and data are shown in Supplementary Figs. S1–S2. (**c**) Immunoblot analyses of CHOhm1 cells reveal both monomeric bands (around 90 kD) and multimeric bands (around 180 and 270 kD) of K_v_7.2 proteins. For clarity, cropped gel images are shown. Full-length gels can be found in Supplementary Fig. S7,a. (**d**) Representative recordings after subtraction of leak currents. Leak current was defined as non-voltage-dependent current from GFP-transfected cells. (**e**) Average peak current densities at all voltage steps. *p < 0.05, ***p < 0.005 based on one-way ANOVA Fisher’s test. (**f**) Average peak current densities at -20 mV (left) and + 20 mV (right). p values are computed from one-way ANOVA Tukey test. (**g**) Normalized conductance (G/Gmax) at all voltage steps. (**h**) Activation time constant (τ) at + 20 mV. The number of GFP-cotransfected cells that were recorded without diC8-PIP_2_: K_v_7.2 WT (n = 12), L_2_03P (n = 17), L268F (n = 17), K552T (n = 13), or R553L (n = 13). The number of GFP-cotransfected cells that were recorded with diC8-PIP_2_: K_v_7.2 WT (n = 11), L_2_03P (n = 14), L268F (n = 13), K552T (n = 11), or R553L (n = 11). Data shown represent the Ave ± SEM.

To test if EE variants within the mutation hotspots disrupt key functional protein domains of K_v_7.2, we selected four EE mutations which have not been previously characterized: L203P at the voltage-sensing S4^23^, L268F at the pore loop^26^, and K552T and R553L at helix B^22,24^ (Fig. 3a,b). To determine their effects on voltage-dependent activation of homomeric K_v_7.2 channels, we performed whole-cell patch clamp recording in Chinese hamster ovary (CHOhm1) cells, which display very low expression of endogenous K^+^ channels and depolarized resting membrane potential of −10 mV^12,33^. Application of depolarizing voltage steps from −100 to +20 mV in GFP-transfected CHOhm1 cells produces very little voltage-dependent currents that reverse around −26 mV^12,34^. In contrast, the same voltage steps in cells transfected with GFP and K_v_7.2 wild-type (WT) generated slowly activating voltage-dependent outward K^+^ currents that reached peak current densities of 17.3 ± 1.1 pA/pF at +20 mV (Fig. 3d,e, Supplementary Fig. S1). The average V_1/2_ of WT channels (−26.8 ± 2.1 mV) was similar to the previously published value of −25 ± 1.9 mV^35^. Consistent with increased outward K^+^ current, cell expressing K_v_7.2 displayed hyperpolarized resting membrane potential (−35.5 ± 1.1 mV) and reversal potential (−38.8 ± 1.9 mV) (Supplementary Tables S4–5.

Cells expressing GFP and K_v_7.2-L203P produced K^+^ currents with a large depolarizing shift in their voltage dependence and V_1/2_ and increased activation time constants, decreasing peak current densities at voltage steps up to 0 mV. These cells also displayed depolarizing resting membrane potential (−26.8 ± 2.0 mV) and reversal potential (−22.1 ± 1.2 mV) (Fig. 3d–f, Supplementary Fig. S1, Supplementary Tables S4–5. Surprisingly, their peak current density at +20 mV was larger (27.6 ± 1.88 pA/pF) than that of WT channels despite their slower activation kinetics (Fig. 3c–e, Supplementary Fig. S1). The L268F mutation in the pore loop decreased outward K^+^ currents through K_v_7.2 channels but not their protein level (Fig. 3c–f, Supplementary Fig. S1). While the R553L mutation in distal helix B had no effect on K_v_7.2 channels, the K552T mutation reduced both protein and current expression (Fig. 3c–f, Supplementary Fig. S1). The L268F and K552T mutations did not alter voltage-dependence, activation kinetics, and reversal potential of K_v_7.2 currents (Fig. 3g,h, Supplementary Tables S4–5).

### PIP_2_-induced potentiation of K_v_7.2 current is blocked by selected EE variants

PIP_2_ is a critical cofactor required for the opening of K_v_7 channels^14,16,17,36^ and is proposed to bind to the intracellular side of S4, the S2-S3 and S4-S5 linkers, and intracellular region from pre-helix A to the helix B-C linker^11–14,28,36–38^. Therefore, we next tested if selected EE mutations alter gating modulation of K_v_7 channels by PIP_2_. Previous studies have shown that the activation of K_v_7 channels is far from saturated by the endogenous membrane level of PIP_2_^39^ and that supplying exogeneous PIP_2_ can enhance single-channel open probability and whole-cell current densities of homomeric K_v_7.2 channels^12,14,37,40^.

Indeed, inclusion of diC8-PIP_2_ (100 μM) in the intracellular pipette solution increased K^+^ currents through K_v_7.2 WT channels by 2-fold and caused a modest left shift in voltage-dependence (Fig. 3d–f, Supplementary Figs. S1–2) as previously shown^12,14,37^. Surprisingly, all selected EE mutations abolished diC8-PIP_2_-induced potentiation of K_v_7.2 channels and hyperpolarizing shift in their voltage dependence, resulting in a significant reduction in their current densities compared to WT channels in the presence of diC8-PIP_2_ (Fig. 3d–f, Supplementary Figs. S1–2, Supplementary Table S5).

To increase cellular PIP_2_ levels, we transfected phosphatidylinositol-4-phosphate 5- kinase (PIP5K), which catalyzes the formation of PIP_2_ via the phosphorylation of phosphatidylinositol-4-phosphate^41^. Consistent with previous reports^11,40,42^, co-expression of PIP5K increased K^+^ currents through K_v_7.2 WT channels with a hyperpolarizing shift in their voltage dependence. Consistent with the recording with diC8-PIP_2_ inclusion (Fig. 3), this effect was absent in K_v_7.2 channels containing L268F, K552T, and R553L variants (Supplementary Fig. S3), indicating that these mutations abolished current potentiation of K_v_7.2 channels upon increasing cellular PIP_2_ levels.

### Modeled K_v_7.2 structure and molecular dynamics simulation suggest that selected EE mutations reside in PIP_2_ binding regions of K_v_7.2

**Figure 4:**
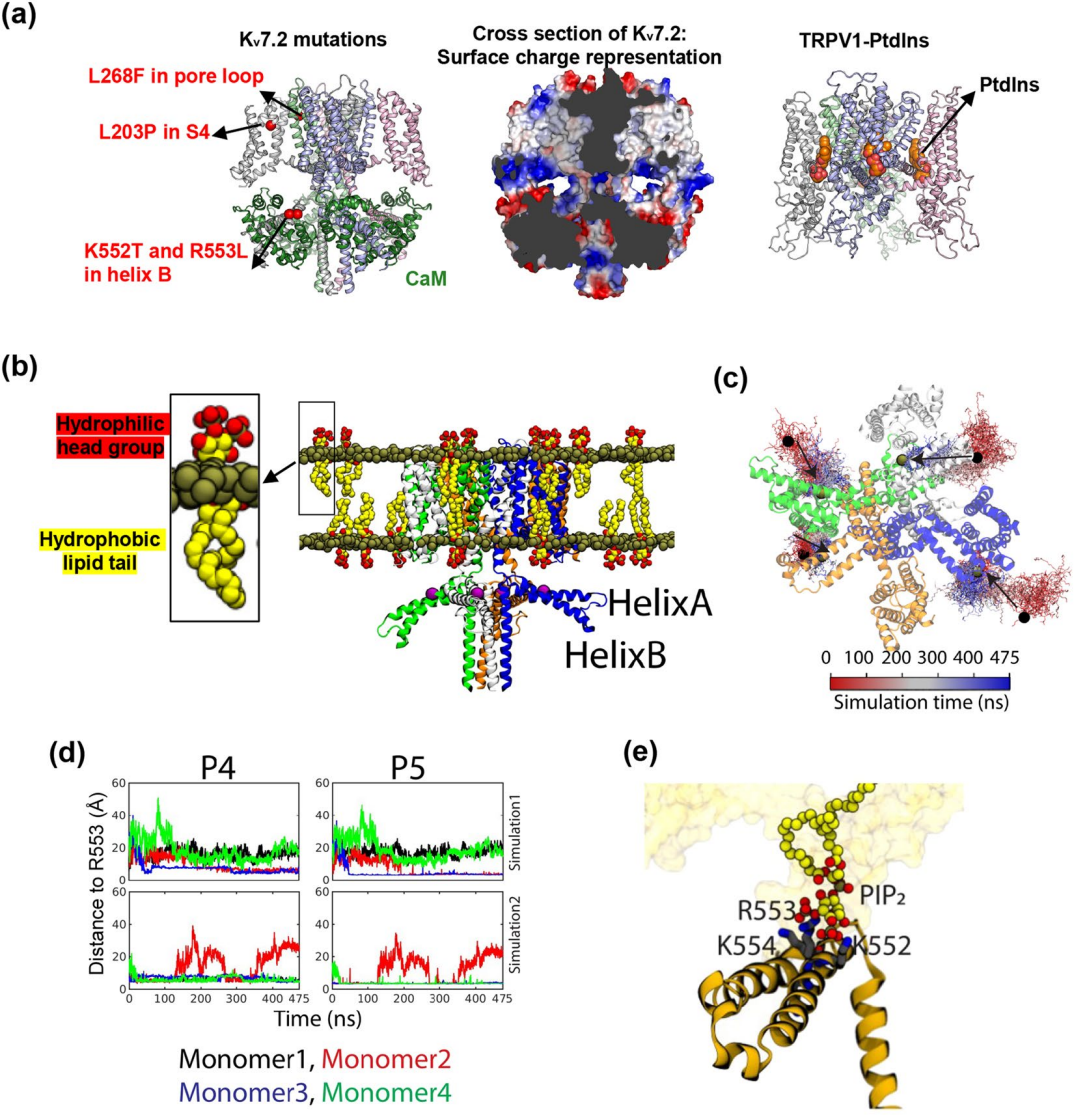
Modeled K_v_7.2 structure and molecular dynamics simulation suggest that selected EE mutations reside in PIP_2_ binding regions of K_v_7.2. (**a**) Homology structure of K_v_7.2 from Fig. 3a and its surface charge representation were compared side-by-side with the cartoon representation of the TRPV1 structure in nanodiscs (PDB: 5irz)^43^. The cross-section view of K_v_7.2 with electrostatic surface showing basic surface in blue, acidic surface in red and nonpolar surface in white. The surface charge representation reveals the nonpolar cavities between the voltage-sensor and the pore domain and indicates that parts of the voltage-sensor, S6, pre-helix A, helix B and helix B-C linker form two basic pockets. (**b**) The open-state conformation of K_v_7.2 channel was embedded in the lipid bilayer. An R553 residue from the helix B of each subunit is represented as a purple sphere. Each subunit is labeled in green, blue, light grey or orange. (**c**) Sample trajectory of PIP_2_ in the modeled K_v_7.2 structure viewed from the bottom (cytoplasmic) side in Fig. 4b. The initial locations of PIP_2_ at the beginning of the molecular dynamics (MD) simulations are indicated in red, and the final positions of PIP_2_ are labeled in blue. The arrow shows the direction of PIP_2_ diffusion in MD simulations. (**d**) The distance between two phosphate groups of PIP_2_ (P4 and P5) and the R553 residue in helix B of K_v_7.2 resulted from two simulations. In the first stimulation, 2 out of 4 K_v_7.2 monomers (monomers 2 and 3) displayed close contact with P4 and P5 of PIP_2_. In the second simulation, 3 out of 4 monomers (monomers 1, 3, and 4) interacted with P4 and P5 of PIP_2_. (**e**) Representative snapshot of the PIP_2_ interacting with K552-R553-K554 in distal helix B of K_v_7.2 (Supplementary Video 1). The confirmation of non-equilibrium simulation and the stability of the modeled open-conformation K_v_7.2 channel structure is shown in Supplementary Fig. 4.

To investigate if selected EE mutations are located in PIP_2_-binding regions, we compared our modeled K_v_7.2 structure bound to CaM and the published structure of TRPV1 channel embedded in lipid nanodiscs with phosphatidylinositol bound (PDB: 5irz) (Fig. 4a)^43^. In the modeled K_v_7.2 structure, the voltage-sensor (S1-S4) and the pore domain of K_v_7.2 form the hydrophobic cavity where L203 and L268 are located (Fig. 4a). Similar to the binding of phosphatidylinositol to TRPV1 channel (Fig. 4a)^43^, the fatty acid tails of amphiphilic PIP_2_ are most likely embedded in this hydrophobic cavity of K_v_7.2 where L203P and L268F mutations reside. Furthermore, the bottom of the voltage-sensor (S1-S4) together with pre-helix A, helix B and the helix B-C linker of K_v_7.2 form a highly basic environment favorable for binding the phosphate headgroup of PIP_2_ (Fig. 4a), consistent with previous studies in K_v_7.1^28^.

To test if PIP_2_ interacts with K552 and R553 in distal helix B, we performed molecular dynamics (MD) simulation. We constructed a homology model of the CaM-bound closed state conformation of K_v_7.2 using the structure of K_v_7.1 (PDB: 5VMS)^28^ as a template, and employed targeted MD to model the open-state conformation of K_v_7.2 in the explicit lipid bilayers containing 1-palmitoyl-2-oleoyl-sn-glycero-3-phosphocholine (POPC) and PIP_2_ lipids. To extensively sample the lipid-protein interactions, we constructed two independent simulation systems, each containing seven PIP_2_ molecules randomly placed around K_v_7.2 without CaM in both outer and inner membrane leaflets (~2.2% PIP_2_) (Fig. 4b). Within the time frame of the simulations, PIP_2_ molecules diffused from the periphery of the K_v_7.2 structure towards its central region (Fig. 4c).

To examine PIP_2_ binding to helix B, we measured the distance between the center of mass of R553 from each monomer and that of the phosphate groups at position 4 and 5 of PIP_2_ (Fig. 4d). Binding of PIP_2_ molecules towards R553 was observed in 2 out of 4 monomers in the first simulation and in 3 out of 4 monomers in the second simulation (Fig. 4d). In both simulations, PIP_2_ molecules interacted with K552-R553-K554 within 100 ns and remained stably bound throughout the simulations (Fig. 4e, Supplementary Video 1, Supplementary Fig. S4), consistent with previous *in vitro* biochemical studies and molecular docking simulations that demonstrated PIP_2_ binding to the corresponding residues in the C-terminal helix A-B fragments of K_v_7.1^37^. These findings suggest that K552T and R553L mutations are located in helix B of K_v_7.2 that interacts with the phosphate head group of PIP_2_.

To test if selected EE mutations alter PIP_2_ affinity, we examined the K_v_7.2 current decay upon PIP_2_ depletion induced by activation of voltage-sensitive phosphatase (VSP)^11,42,44^. In CHOhm1 cells coexpressing *danio rerio* VSP^11^, the 10s-depolarization step at voltages from +40 mV decreased peak currents of K_v_7.2 channels, reaching the maximal decay of 53.2 ± 4.0% at +100 mV (Supplementary Fig. S5). Current decay of K_v_7.2-K552T was greater than that of WT at +40 mV but was comparable to that of WT from +60 to +100 mV (Supplementary Fig. S5), suggesting that the K552T mutation modestly decreased PIP_2_ affinity to K_v_7.2. Interestingly, the same depolarization steps delayed current decay of K_v_7.2 channels containing L203P, L268F, and R553L mutations (Supplementary Fig. S5), indicating their reduced sensitivity to PIP_2_ depletion.

### Selected EE variants decrease current expression of heteromeric K_v_7 channels and their current potentiation by diC8-PIP_2_

**Figure 5:**
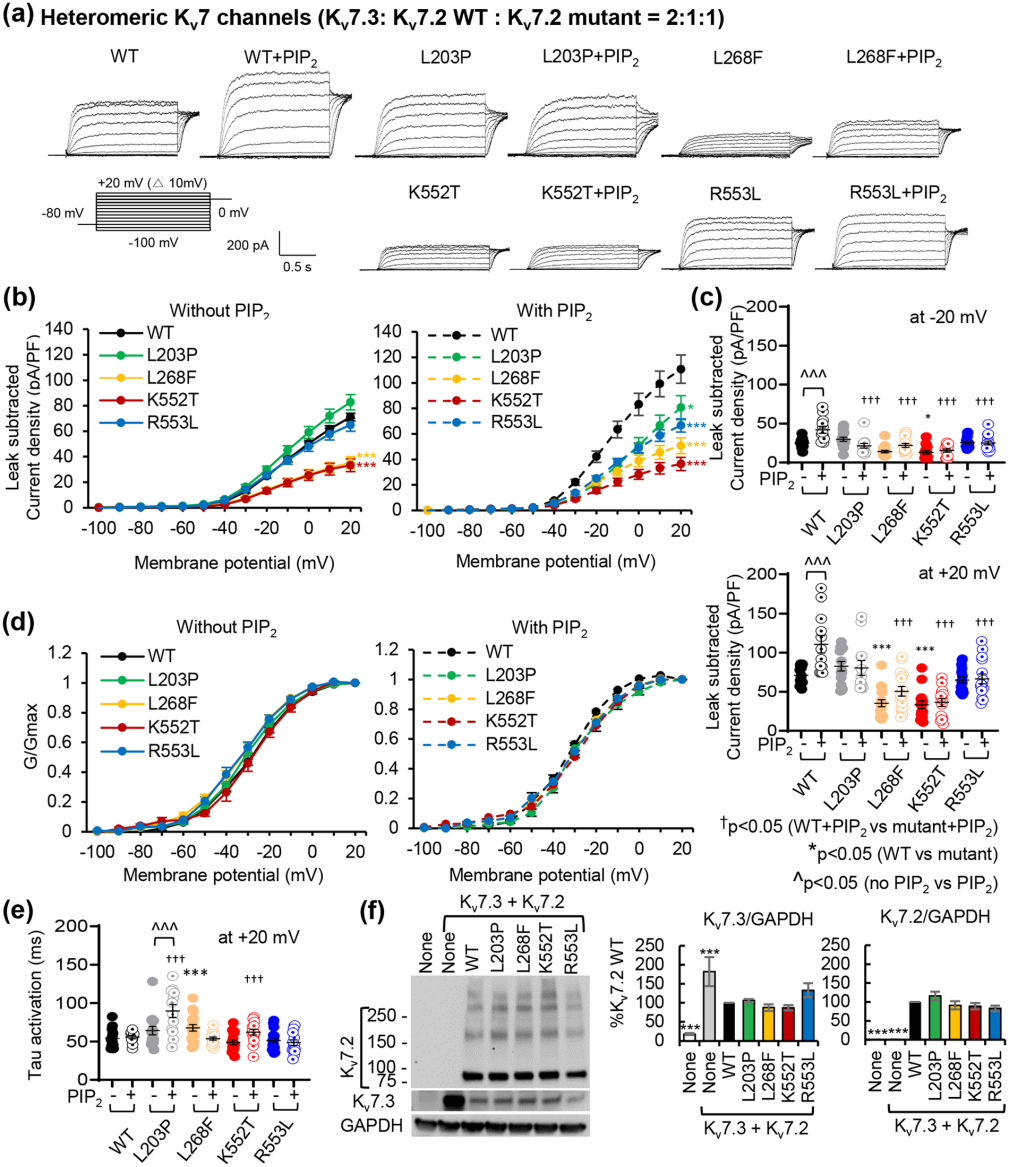
The L268F and K552T mutations decreased current expression of heteromeric K_v_7 channels whereas all selected EE mutations disrupted their current potentiation by diC8-PIP_2_ inclusion. Whole cell patch clamp recordings were measured using the voltage clamp protocol described in Fig. 3 from GFP-positive CHOhm1 cells cotransfected with K_v_7.3 and K_v_7.2 WT (1:1 ratio) or K_v_7.3, K_v_7.2 WT, K_v_7.2 mutant (2:1:1 ratio). The raw current traces and data are shown in Supplementary Figs. S6 and S7. (**a**) Representative leak-subtracted current traces. (**b**) Average leak-subtracted peak current densities at all voltage steps. *p < 0.05, ***p < 0.005 based on one-way ANOVA Fisher’s test. (**c**) Average leak-subtracted peak current densities at -20 mV (top) and at + 20 mV (bottom). p values are computed from one-way ANOVA Tukey test. (**d**) Normalized conductance (G/Gmax) at all voltage steps. (**e**) Activation time constant (τ) at + 20 mV. The number of GFP-cotransfected cells that were recorded without diC8-PIP_2_: K_v_7.2 WT (n = 14), L_2_03P (n = 15), L268F (n = 18), K552T (n = 15), or R553L (n = 15). The number of GFP-cotransfected cells that were recorded with diC8-PIP_2_: K_v_7.2 WT (n = 12), L203P (n = 12), L268F (n = 16), K552T (n = 14), or R553L (n = 15). (**f**) Immunoblot analyses of CHOhm1 cells co-transfected with K_v_7.2 wild-type or mutant and K_v_7.3 reveal both monomeric bands (around 90 kD) and multimeric bands (around 180 kD, 270 kD, and 370 kD) of K_v_7.2 proteins. For clarity, cropped gel images are shown. Full-length gels can be found in Supplementary Fig. S8,b. Data represent the Ave ± SEM. ***p < 0.005.

Since *KCNQ2*-associated EE is an autosomal dominant epileptic syndrome, we repeated voltage-clamp recording in CHOhm1 cells transfected with plasmids for K_v_7.3, wild-type K_v_7.2, and mutant K_v_7.2 at a 2:1:1 ratio as described^35^ (Fig. 5, Supplementary Figs. S6–7). Although the L203P variant induced a large depolarizing shift in voltage-dependence of homomeric channels (Fig. 3d–g), heteromeric L203P mutant channels were indistinguishable from WT channels (Fig. 5a–d). Similar to homomeric channels (Fig. 3), heteromeric channels containing mutations L268F and K552T but not R553L produced significantly less current than WT channels without changing their voltage dependence (Fig. 5a–d, Supplementary Table S5). The L268F variant also increased their activation kinetics (Fig. 5e). None of the tested mutations affected total protein expression of K_v_7.2 and K_v_7.3 (Fig. 5f).

When diC8-PIP_2_ was added in the intracellular pipette, all tested EE mutations significantly decreased current densities of heteromeric channels at +20 mV compared to WT without altering their voltage-dependence (Fig. 5a–d, Supplementary Table S5) and their activation time constant was increased by L203P and K552T mutations (Fig. 5e). Importantly, all tested EE variants abolished PIP_2_-induced current potentiation of heteromeric channels (Fig. 5a–c, Supplementary Figs. S6–7).

### Selected EE variants variably decrease axonal surface expression of heteromeric K_v_7 channels

**Figure 6:**
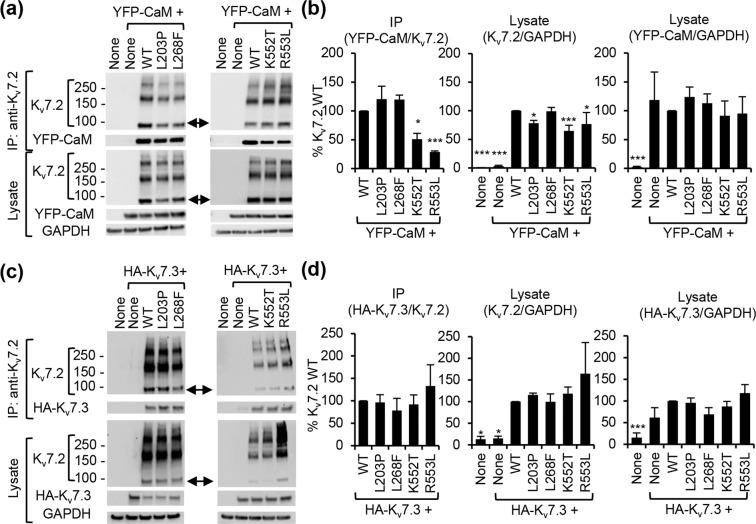
The K552T and R553L mutations reduced CaM binding to K_v_7.2 whereas none of the tested EE mutations affected K_v_7.3 interaction with K_v_7.2. (**a**,**b**) Co-immunoprecipitation of YFP-CaM with wild-type K_v_7.2 (WT) or K_v_7.2 containing EE mutations in the presence of EGTA. (**a**) Representative immunoblots of HEK293T cells expressing K_v_7.2 and YFP-CaM. For clarity, cropped gel images are shown. Full-length gels can be found in Supplementary Fig. S9. (**b**) Quantification of immunoblots: untransfected/None (n = 6), YFP-CaM (n = 6), YFP-CaM cotransfection with K_v_7.2 WT (n = 6), L203P (n = 3), L268F (n = 3), K552T (n = 3), or R553L (n = 3). **(c**,**d)** Co-immunoprecipitation of HA-K_v_7.3 with wild-type K_v_7.2 (WT) or K_v_7.2 containing EE mutations in the presence of EGTA. **(c)** Representative immunoblots of HEK293T cells expressing K_v_7.2 and K_v_7.3. For clarity, cropped gel images are shown. Full-length gels can be found in Supplementary Figs. S9–10. **(d)** Quantification of immunoblots: untransfected cells (None: n = 3), or cells transfected with HA-K_v_7.3 (n = 4), HA-K_v_7.3 and K_v_7.2 WT (n = 4), L203P (n = 3), L268F (n = 3), K552T (n = 4), or R553L (n = 3). GAPDH served as a loading control. Both monomeric K_v_7.2 bands (around 90kD, arrows) and multimeric K_v_7.2 bands (around 180 kD, 270 kD, and 370 kD) are observed in the IP samples and lysate in (**a**,**c**). Data represent the Ave ± SEM (*p < 0.05, ***p < 0.005 against K_v_7.2 WT).

**Figure 7:**
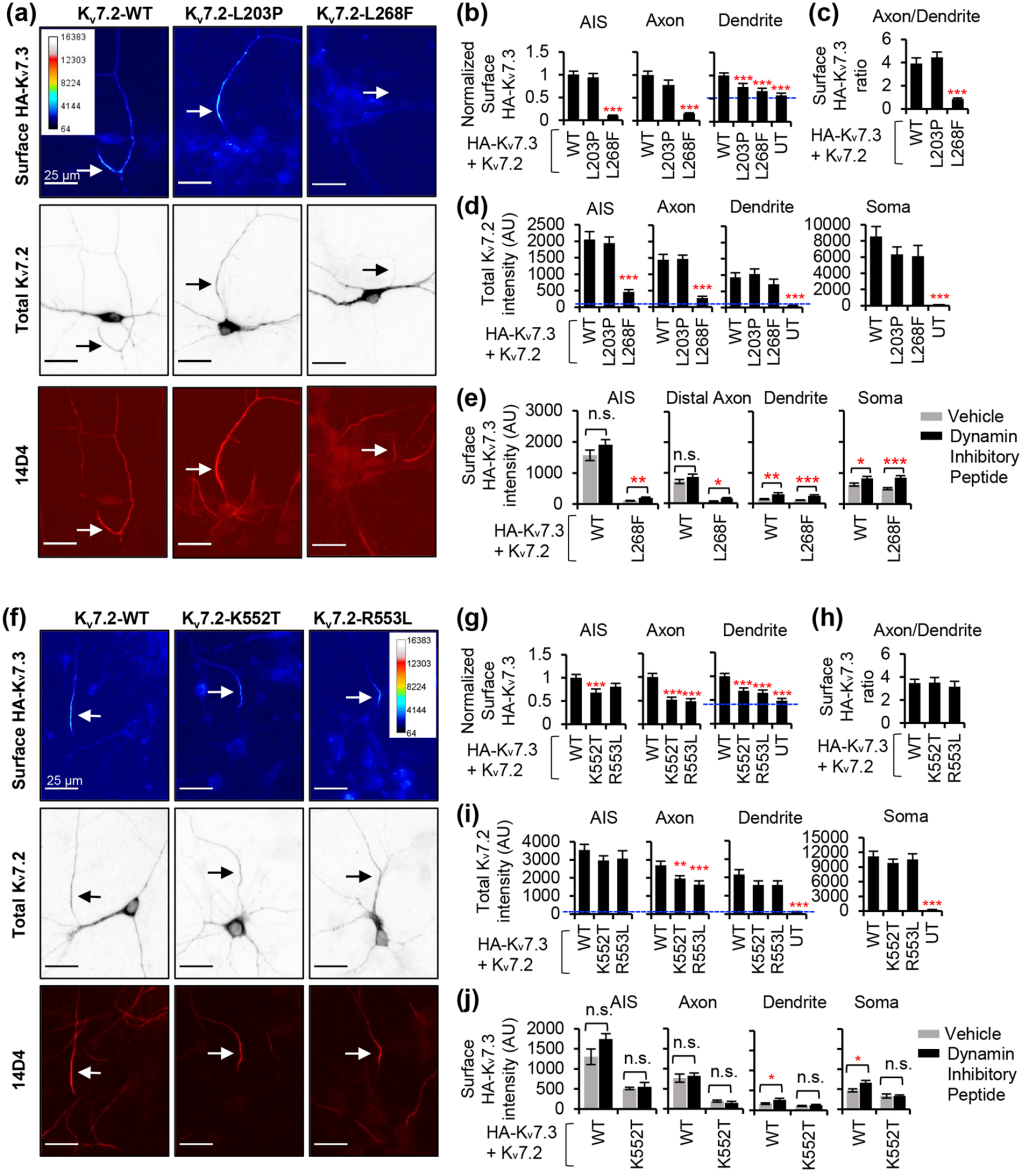
L268F, K552T, and R553L mutations decreased enrichment of heteromeric K_v_7 channels at the axonal surface in cultured hippocampal neurons. Immunostaining of surface K_v_7.3 and total K_v_7.2 containing an extracellular hemagglutinin epitope (HA-K_v_7.3) in healthy hippocampal neurons cotransfected with K_v_7.2 WT or K_v_7.2 with EE mutations L203P, L268F (**a–e**), K552T, and R553L (**f–j**). **(a**,**f)** Representative images of surface HA-K_v_7.3 (*Upper*) as pseudo-color that display differences in the surface HA intensities from high (red) to low (blue). Total K_v_7.2 (*Middle-inverted gray*) and the AIS identified by antibodies for phospho IκBα-Ser32 (14D4) (*Lower-fluorescence*) are shown in same neurons. Arrows mark the AIS. Scale bars: 25 μm. (**b**,**g**) Normalized background-subtracted mean intensities of surface HA fluorescence from neurons expressing WT and EE mutant HA-K_v_7.3/K_v_7.2 channels. The number of transfected neurons that were analyzed in Fig. 7b: WT (n = 41), L203P (n = 39), L268F (n = 34), untransfected (UT) (n = 20). The number of transfected neurons that were analyzed in Fig. 7g: WT (n = 42), K552T (n = 34), R553L (n = 21), untransfected (UT) (n = 19). The raw data from 3 independent experiments are shown in Supplementary Fig. S11. (**c**,**h**) Surface HA intensity ratio at distal axon over dendrite. (**d**,**i**) Background-subtracted mean intensities of total K_v_7.2 fluorescence in K_v_7.2-transfected neurons and untransfected neurons (UT, blue dotted lines). The number of transfected neurons that were analyzed in Fig. 7d: K_v_7.2 WT (n = 14), L203P (n = 15), L268F (n = 14), UT (n = 14). The number of transfected neurons that were analyzed in Fig. 7i: K_v_7.2 WT (n = 17), K552T (n = 23), R553L (n = 14), UT (n = 13). **(e**,**j**) Background-subtracted mean intensities of surface HA fluorescence from the transfected neurons treated with vehicle (CTL) control or dynamin inhibitory peptide (DIP). The number of transfected neurons that were analyzed in Fig. 7e: WT + CTL (n = 14), WT + DIP (n = 13), L268F + CTL (n = 8), L268F + DIP (n = 8). The number of transfected neurons that were analyzed in Fig. 7j: WT + CTL (n = 8), WT + DIP (n = 13), L268F + CTL (n = 6), L268F + DIP (n = 7). Data represent the Ave ± SEM (*p < 0.05, **p < 0.01, ***p < 0.005 against WT channels).

The physiologically relevant current through K_v_7 channels is controlled by both their function and expression at the neuronal plasma membrane. Given that K_v_7.2 interaction with CaM and K_v_7.3 are critical for axonal surface expression of K_v_7 channels^9,45^, we next tested if selected EE variants of K_v_7.2 affect interaction with CaM and K_v_7.3 and axonal targeting of K_v_7 channels (Figs. 6–7, Supplementary Figs. S9–11). Coimmunoprecipitation assay in HEK293T cell lysate^12,45^ revealed that the K552T and R553L mutations in helix B decreased K_v_7.2 binding to YFP-tagged CaM whereas the mutations including L203P in S4 and L268F in the pore loop had no effect (Fig. 6a,b). None of the tested mutations affected K_v_7.2 interaction with K_v_7.3 (Fig. 6c,d). Total K_v_7.2 expression was also reduced by the L203P and K552T variants in cells co-expressing CaM but not K_v_7.3 (Fig. 6).

To test if selected EE mutations of K_v_7.2 affect surface density of K_v_7 channels, we transfected rat dissociated hippocampal cultured neurons with K_v_7.3 containing an extracellular HA epitope (HA-K_v_7.3) and performed surface immunostaining of HA-K_v_7.3 as described^9,12,45^ (Fig. 7, Supplementary Fig. S11). In cultured neurons, transfection of HA-K_v_7.3 alone yields negligible surface expression of HA-K_v_7.3^9^. However, co-transfection of K_v_7.2 WT results in robust HA-K_v_7.3 expression on the plasma membrane of the AIS and distal axons compared to the soma and dendrites (Fig. 7a,b)^9,12,45^, resulting in a surface fluorescence “Axon/Dendrite” ratio of 3.9 ± 0.49 (Fig. 7c).

Although the L203P mutation in S4 did not affect surface and total expression of HA-K_v_7.3/ K_v_7.2 channels (Fig. 7a–d), the L268F mutation in the pore loop abolished their preferential enrichment at the axonal surface by severely decreasing their axonal surface density (surface Axon/Dendrite ratio = 0.85 ± 0.10, Fig. 7a–c) and also reduced intracellular K_v_7.2 expression in the axon (Fig. 7d). The K552T and R553L mutations in helix B significantly reduced surface expression of heteromeric channels in both distal axon and dendrites (Fig. 7f–i), resulting in similar surface Axon/Dendrite ratios as the WT channels (Fig. 7h).

Disruption of CaM binding to K_v_7.2 has been shown to impair axonal enrichment of K_v_7 channels by inhibiting their trafficking from the endoplasmic reticulum (ER)^45^. The ability of the L268F mutation to impair axonal K_v_7 surface expression without affecting K_v_7.2 binding to CaM or K_v_7.3 (Figs. 6, 7a–d) suggests a different mechanism. To test if the L268F mutation reduces axonal enrichment of K_v_7 channels by increasing their endocytosis, we used dynamin inhibitory peptide (DIP, 50 μM) which blocks dynamin-dependent endocytosis in cultured hippocampal neurons^46^. The DIP treatment for 45 min induced a small increase in surface HA-K_v_7.3/ K_v_7.2 WT and L268F mutant channels in the soma and dendrites but not axons (Fig. 7e,j), indicating their basal endocytosis in somatodendritic membrane. Although the DIP treatment had no effect on K552T mutant channels, the same treatment modestly increased axonal surface expression of L268F mutant channels (Fig. 7e,j). However, this increase did not reach the axonal level of WT channels (Fig. 7e), suggesting that increased endocytosis is not the main cause for reduced axonal surface expression of L268F mutant channels.

## Discussion

In this study, we investigated the pathogenetic mechanisms underlying *de novo* EE mutations of K_v_7.2. Visual inspection in K_v_7.2 primary sequence has suggested the enrichment of EE variants at S4, the pore domain from S5 to S6, and helices A and B^12,25,47^. Clustering of epilepsy mutations in the ion transport domain of K_v_7.2 has also been detected by identifying its variation-intolerant genic sub-regions^48^. Our novel MHF statistical algorithm interpreted in the context of modeled K_v_7.2 atomic structure (Figs. 1–2) supports these earlier observations. We discovered that “severe or EE” missense variants cluster at S4, the pore loop that contains the selectivity filter, S6, helix B, and the helix B-C linker of K_v_7.2 (Fig. 1). A recent study by Goto *et al*., reported that the EE missense variants cluster at the pore domain, S6, and pre-helix A of K_v_7.2^49^. The regional differences in mutation clusters between our study and Goto et al., could be attributed to the use of different algorithms and databases (ExAC and GnomAD) as sources for non-pathogenic mutations. Nonetheless, both studies identified the pore domain and S6 as hotspots of EE variants, supporting the functional importance of these regions.

However, sequence variant interpretation from the prediction algorithms should be used carefully. The presence of both gain-of-function and loss-of-function EE variants in S4^47,50–52^ suggest that it is not straight forward to predict the genotype-phenotype correlation of EE. In addition, both EE and BFNE variants exist in each of the identified hotspots and even at the same codon^18,49^, suggesting that different amino acid substitutions at the same residue may cause diverse effects on K_v_7 channels and the clinical severity of epilepsy. Although this challenge has been addressed recently by Percent Accepted Mutation (PAM)30 algorithm based on amino acid substitution in evolution^49^, the *in vivo* impact of a mutation is difficult to predict in patients due to their variable exposures to genetic and environmental factors. Thus, the use of multiple *in-silico* tools and comprehensive experimental analyses of epilepsy variants are needed to understand their effects on K_v_7 channels *ex vivo* and *in vivo*.

Our functional characterization of *de novo* EE variants selected from the mutation hotspots revealed that each mutation impaired the function of its associated protein domain within K_v_7.2. The L203P mutation in the main voltage sensor S4 induced a large depolarizing shift in voltage-dependence and slowed activation kinetics of homomeric K_v_7.2 channels (Fig. 3) but had no effect on heteromeric channels (Fig. 5). In contrast, the L268F mutation in the pore loop decreased current densities of both homomeric and heteromeric channels without affecting their voltage dependence (Figs. 3, 5). K552T and R553L mutations in CaM-binding helix B decreased the interaction between CaM and K_v_7.2 (Fig. 6), which is shown to play critical roles in M-current expression and inhibition of hippocampal neuronal excitability^53^. Current suppression of homomeric channels is a common feature of EE variants of *KCNQ2*^54^. Given the overlapping distribution of K_v_7.2 and K_v_7.3 throughout the hippocampus and cortex^4^, the dominant negative functional effects of L268F and K552T variants on heteromeric channels (Figs. 3, 5) are expected to induce neuronal hyperexcitability and may underlie severe symptomatic EE with drug-resistant seizures, psychomotor delay, and profound intellectual disability^22,26^.

Interestingly, our modeled K_v_7.2 structure revealed that the distal helix B and helix B-C linker come together with pre-helix A to form a positively charged surface close to the voltage sensor S1-S4 and the base of S6 (Fig. 2). Mutations of basic amino acid residues including H328C, R325G, and R333W at pre-helix A and R560W at the helix B-C linker of K_v_7.2 have been shown to impair regulation of K_v_7.2 currents by PIP_2_^11,12,14^, which couples voltage sensor activation to the opening of the gate^28,36^. Our MD simulations revealed that K552 and R553 in distal helix B bind to the negatively charged head group of PIP_2_ (Fig. 4). Importantly, K552T and R553L mutations impaired current enhancement of both homomeric and heteromeric channels upon acute or tonic increase in PIP_2_ (Figs. 3, 5, Supplementary Fig. S3), suggesting that these mutant channels cannot respond to the changes in cellular PIP_2_. Since stable binding of CaM to K_v_7.2 is crucial for PIP_2_ modulation of neuronal K_v_7 channels^33^, a decrease in CaM binding (Fig. 6) may also contribute to the loss of PIP_2_-induced current enhancement of K552T and R553L mutant channels (Figs. 3, 5).

The impairment of PIP_2_-induced current enhancement of L268F mutant channels was unexpected (Figs. 3, 5–7) because it is unlikely for the hydrophobic L268 to bind a negatively charged head group of PIP_2_. A comparison between modeled K_v_7.2 structure and TRPV1 structure (Fig. 4a) suggests that the amphiphilic chains of PIP_2_ may extend to the hydrophobic cavity created by the voltage-sensors S1-S4 and the pore domain of K_v_7.2. We speculate that the L268F mutation at this hydrophobic interface impair K_v_7.2 interaction with PIP_2_. Furthermore, analogous residue for L268 in the bacterial KcsA structure can secure the proper opening size of the pore^55^. Therefore, it is also possible that the L268F mutation may disrupt PIP_2_-dependent coupling to the pore opening^36^.

Several studies including our own have investigated PIP_2_ affinity of K_v_7 function by inclusion of diC8-PIP_2_ in the intracellular pipette solution^12,14,33,37^. However, caution must be exercised in interpreting their results. Potentiation of K_v_7.2-L203P current by tonic elevation of cellular PIP_2_ upon PIP5K expression but not acute application of diC8-PIP_2_ (Fig. 3, Supplementary Fig. S3), suggest that diC8-PIP_2_ inclusion may not readily potentiate the mutant channels that displayed very slow activation kinetics (Fig. 3). Furthermore, the loss of diC8-PIP_2_-induced current potentiation can be either due to decreased PIP_2_ affinity or saturated level of interaction with PIP_2_ at low PIP_2_ concentration. We found that the K552T mutation modestly weakens PIP_2_ affinity, whereas other mutant channels were resistant to PIP_2_ depletion (Supplementary Fig. S5). Considering multiple proposed PIP_2_ binding sites in K_v_7.2 including S2-S3 and S4-S5 linkers, pre-helix A, helix B, and helix A-B and helix B-C linkers^11–15^, selected EE mutations may cause conformational change that weakens or enhances PIP_2_ affinity to other regions within K_v_7.2. As Suh and Hille (2008) pointed out^56^, it is not straight forward to determine PIP_2_ affinity of mutant channels by assessing their currents after manipulation of PIP_2_ level. Nonetheless, the lack of current potentiation upon increasing cellular or exogenous PIP_2_ (Fig. 3, Supplementary Fig. S3) and the location of the EE mutated residues in a region of K_v_7.2 that binds to fatty acid tails or polar headgroups of PIP_2_ (Fig. 4) strongly suggest that there are multiple ways by which the selected EE variants may influence PIP_2_ interaction with K_v_7 channels and reduce their currents.

Our investigation of selected EE variants on neuronal expression of K_v_7 channels revealed that K552T and R553L mutations in helix B reduced enrichment of K_v_7 channels at the axonal surface (Fig. 7), supporting previous observations that the degree of CaM interaction with K_v_7.2 correlates with the overall amount of K_v_7 channels at the axonal surface^12,45^. Unexpectedly, the L268F mutation at the pore loop severely decreased both surface and intracellular expression of heteromeric channels in axons without affecting K_v_7.2 binding to CaM or K_v_7.3 (Figs. 6–7), demonstrating the importance of studying K_v_7 expression in neurons. Decreased axonal expression of K_v_7.2-L268F and minor effects of endocytosis inhibition (Fig. 7) suggest that a severe reduction of L268F mutant channels at the axonal surface is caused by a CaM- and endocytosis-independent mechanism. Given that misfolded membrane proteins are retained in the ER for chaperone-assisted refolding^57^, the L268F mutation may cause a folding defect that facilitates ER retention and disrupts forward trafficking of heteromeric channels to the axon.

**Figure 8:**
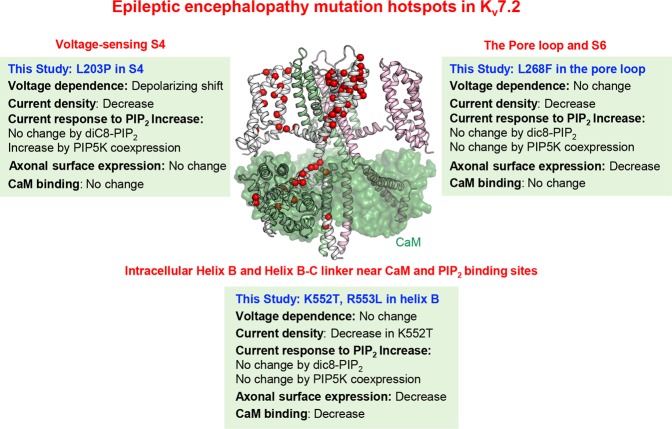
A summary for pathogenetic mechanisms of epilepsy mutation hotspots in K_v_7.2. Our functional characterization of EE mutations selected from the epilepsy mutation hotspots of K_v_7.2 show that they not only disrupt the functions of key protein domains they reside in, but also reduce PIP_2_-dependent current potentiation and axonal surface expression of K_v_7 channels. The missense EE mutations are highlighted with red spheres on the C-alpha atoms of one subunit on the modeled tetrameric human K_v_7.2 structure (ribbons) in complex with four CaM subunits (transparent green surfaces). The list of these EE variants can be found in Supplementary Table S7.

Taken together, we identified EE mutation hotspots in K_v_7.2 and discovered that each variant selected from these hotspots impairs the function of its associated protein domain and displays a combination of defects in voltage- and PIP_2_-dependent activation and axonal expression of K_v_7 channels (Fig. 8). Such combinations of defects may decrease K_v_7 current and its ability to inhibit neuronal excitability in neonatal brain^5,12^, as conditional deletion of K_v_7.2 during embryonic development results in hippocampal and cortical hyperexcitability and spontaneous seizures in mice^7^. Continued optimization of prediction algorithms and experimental interrogations to understand pathophysiology of K_v_7-associated EE will aid the development of better therapeutic strategies for this disease.

## Materials and Methods

### The resampling statistical algorithm

A resampling algorithm titled Mutation Hotspot Finder (MHF) was developed to search for mutation clusters that localize to the functional domains in human K_v_7.2 (GenBank: NP_742105.1). The complete MHF algorithm can be found in GitHub repository (https://github.com/jerrycchen/MutationHotspotFinder). The functional domains were annotated based on multiple published sources^3,28,30,32^ and the RIKEE database (www.rikee.org). Briefly, the MHF algorithm compares the observed numbers of mutations against the expected numbers of mutations, and computes corresponding statistical significance through bootstrapping within each pre-specified protein functional domains. The following sections explain the MHF algorithm in detail.

The $S$ denote the set of all observed single amino acid mutations for the whole sequence of protein $X$ (e.g. the whole sequence of K_v_7.2), or the subset of the sequence of protein $X$ (e.g. the intracellular C-terminal tail of K_v_7.2). $|S|=length(S)$ indicates the number of unique mutations in $S.$

The ${D}_{j},\,j\in \{1,\,2,\ldots ,J\},$ denote the number of mutations in $S$ that fall into the functional domain $J$ of protein sequence $X$. Among 14 functional domains in K_v_7.2 (Supplementary Table S2), ${D}_{1}$ is the number of mutations in the S1, and ${D}_{2}$ is the number of mutations in the S2, and etc. Among 9 functional domains in intracellular K_v_7.2 C-terminal tail (Supplementary Table S2), ${D}_{1}$ is the number of mutations in the pre-Helix A, and ${D}_{2}$ is the number of mutations in the Helix A, and etc.

The MHF algorithm assumes that mutations are equally observed at each amino acid position within functional domains when there is no further association between the mutations and the domains. Due to this null hypothesis^58^, the application of MHF algorithm is restricted to single amino acid mutations, and is not suitable for mutations outside of the coding sequence as well as nonsense or frameshift mutations that delete one or more function domains. Under such assumption, we can randomly draw samples (i.e. bootstrap), with size = $|S|$, from the sequence $X$ to construct the bootstrapped “mutation sets” in order to simulate the distribution of mutations.

For iteration $\,k$ of bootstrapping, the $\tilde{S}(k)$ denote the bootstrapped mutation set where $k\in \{1,\,2,\ldots ,K\}$. In the context of this paper, we ran 10,000 iterations of bootstrapping (i.e. $K=10,000$). The ${\tilde{D}}_{j}(k)$ is defined as the number of mutations in $\tilde{S}(k)$ that fall into the functional domain $j$ of protein sequence $X$. The empirical expected number of mutations within each domain $j$ was constructed from

$${\hat{E}}_{j}=\frac{1}{K}\sum _{k}{\tilde{D}}_{j}(k).$$

The empirical *P*-values (${\hat{P}}_{j}$) were computed from a right-tailed test to measure the level of statistical significance on the proportion of the bootstrapped mutation sets that had more mutations than the observed mutation set $S$ at each individual protein functional domain.

$${\hat{P}}_{j}=\frac{1}{K+1}\sum _{k}\{I[{\tilde{D}}_{j}(k)\ge {D}_{j}]+1\},$$

where $I(\cdot )$ is the Indicator function.

The computed *P*-values were adjusted for multiple comparisons ($J$ times) using Bonferroni’s correction. Mutations were visualized and mapped to K_v_7.2 and K_v_7.3 primary structures with MutationMapper (http://www.cbioportal.org/mutation_mapper.jsp Mapper). Fisher’s Exact Test was implemented using the standard fisher.test() function in R (https://stat.ethz.ch/R-manual/R-devel/library/stats/html/fisher.test.html).

### Structure modeling and visualization

The S1-S6 sequence of K_v_7.2 (R75-Q326) was threaded to the cryo-EM structure of *Xenopus laevis* K_v_7.1 bound to CaM (PDB: 5VMS)^28^. The loops of K_v_7.2 (E86-W91 and K255-T263) were rebuilt in FoldIt (https://fold.it/portal). The structure was relaxed in Rosetta software (https://www.rosettacommons.org/software) using two rounds of rotamer sampling followed by side chain and backbone minimization, ending with minimization of all degrees of freedom while maintaining C4 symmetry. The lowest scoring decoy with Root mean square deviation (RMSD) < 2.0 Å was chosen as the final model. The amino acid residues mutated in BFNE and EE are indicated in the Rosetta-based model.

To model the interaction between CaM and K_v_7.2 helices A and B, the helix A sequence of K_v_7.2 (E322-V367) was threaded to the crystal structure of chimeric K_v_7.3 helix A - K_v_7.2 helix B in complex with Ca^2+^-bound CaM (PDB: 5J03)^32^. The structure was relaxed with Rosetta using two rounds of sequential rotamer, side chain and backbone minimization, followed by rigid body minimization. Mutations were made to the model in Rosetta followed by sequential rotamer, side chain, backbone, and rigid body minimization. The binding energy was calculated from 20 simulations. Structures were visualized using PyMOL 2.0 (Schrödinger, LLC).

### DNA Constructs and mutagenesis

EYFP-hCaM was a gift from Dr. Emanuel Strehler (Addgene plasmid # 47603). The plasmid pIRES-dsRed-PIPKIγ90 was a gift from Dr. Anastasios. Tzingounis (University of Connecticut) and was previously described^42^. Plasmids pcDNA3 with *KCNQ*3 cDNA (GenBank: NM004519) encoding K_v_7.3 (GenBank: NP_004510.1), HA-K_v_7.3, and *KCNQ*2 cDNA (GenBank: Y15065.1) encoding K_v_7.2 (GenBank: CAA 75348.1) have been previously described^9,12,45^. Compared to the reference sequence of K_v_7.2 (GenBank: NP_742105.1), this shorter isoform lacks 2 exons which do not harbor pathogenic variants to date. However, the amino acid numbering in the manuscript conforms to the reference sequence of K_v_7.2 for clarity. Epileptic encephalopathy mutations (L203P, L268F, K552T, R553L) were generated using the Quik Change II XL Site-Directed Mutagenesis Kit (Agilent).

### Electrophysiology

Whole cell patch clamp recordings in Chinese hamster ovary (CHO hm1) was performed as described^12^. To express homomeric K_v_7.2 channels, cells were transfected with pEGFPN1 (0.2 μg) and pcDNA3-K_v_7.2 WT or mutant (0.8 μg). To express K_v_7.2 channels and PIP5K, cells were transfected with pEGFPN1 (0.2 μg), pIRES-dsRed-PIPKIγ90 (0.45 ug, a kind gift from Dr. A. Tzingounis, U. Conn^42^), pcDNA3-K_v_7.2 WT or mutant (0.45 μg). For the negative control for the PIP5K experiment, the cells were transfected with pEGFPN1 (0.65 μg) and pcDNA3-K_v_7.2 WT or mutant (0.45 μg). To express heteromeric channels, cells were transfected with pEGFPN1 (0.4 μg), pcDNA3-K_v_7.3 (0.8 μg), pcDNA3-K_v_7.2 WT (0.4 μg), or pcDNA3-K_v_7.2 WT or mutant (0.4 μg). Leak-subtracted current densities (pA/pF), normalized conductance (G/Gmax), and channel biophysical properties were computed as described^12,35^ with the exception that V_1/2_ and the slope factor *k* were calculated as described^35,59^ by fitting the plotted points of G/Gmax with a Boltzmann equation G/Gmax = 1/ {1 + exp (V_0.5_ – V_c_) / *k*}.

To examine the decline of K_v_7.2 current upon activation of Dr-VSP, CHO hm1 cells were transfected with pDrVSP-IRES2-EGFP (0.5 μg) and pcDNA3-K_v_7.2 WT or mutant (0.5 μg). The pDrVSP-IRES2-EGFP plasmid was a gift from Yasushi Okamura (Addgene plasmid # 80333). Voltage-clamp recording of K_v_7.2 current upon depolarization-induced Dr-VSP activation was performed as described^60^ with an external solution containing 144 mM NaCl, 5 mM KCl, 2 mM CaCl2, 0.5 mM MgCl2, 10 mM glucose and 10 mM HEPES (pH 7.4). Patch pipettes (3 – 4 MΩ) were filled with intracellular solution containing 135 mM potassium aspartate, 2 mM MgCl2, 1 mM EGTA, 0.1 mM CaCl2, 4 mM ATP, 0.1 mM GTP and 10 mM HEPES (pH 7.2). Cells were held at -70 mV and 10 s step depolarizations were applied in 20 mV steps from -20 to +100 mV with 2 min inter-step intervals to allow PIP_2_ regeneration. The extent of K_v_7.2 current decay upon Dr-VSP activation during 10 s depolarization was measured as the ratio of current at 10 s over peak current at each voltage step.

### Molecular dynamics simulation

For modeling of open and closed states of K_v_7.2, the closed-state conformation of KCNQ2 in calmodulin-bound form was modeled based on the recent cryo-EM structure of K_v_7.1 (PDB code 5VMS)^28^. Multiple sequence alignment of the template and KCNQ2 sequence was performed by using TCoffee web server (https://www.ebi.ac.uk/Tools/msa/tcoffee/). After the alignment, the homology model of closed-state conformation was built with MODELLER^61^. The stability of the closed-state conformation of K_v_7.2 was tested by performing all-atom molecular dynamics (MD) simulations in explicit lipid bilayer.

In order to model the open-state conformation of K_v_7.2, we performed non-equilibrium MD simulations. Using our stable closed-state conformation of K_v_7.2, we performed 20-ns of Targeted MD (TMD)^62^ simulations in an explicit lipid bilayer. TMD has been shown to drive the conformational changes by gradually minimizing the RMSD of S4-S5 and S6 helices of the closed-state conformation and the target structure which is K_v_1.2/K_v_2.1 in open conformation (PDB: 2R9R)^63^. As major structural changes occur in the pore region of the channel, we applied a restraint (force constant = 250 kcal/mol/Å) on the S4-S5 and S6 helices of each monomer to drive it towards the target state which was defined by a highly homologous K_v_1.2/K_v_2.1 channel in open-state conformation (PDB code 2R9R)^63^. The success of TMD was gauged by measuring the backbone RMSD of S4-S5 and S6 helices with respect to the target (Supplementary Fig. S6). Upon completion of TMD, all the structural restraints were released and the stability of the obtained open-state conformation of K_v_7.2 was tested by performing MD simulations in explicit lipid bilayer (Supplementary Fig. S6).

For MD simulation, the modeled K_v_7.2 without calmodulin was embedded in the lipid bilayer, containing 1-palmitoyl-2-oleoyl-sn-glycero-3-phosphatidylcholine (POPC) and 1-palmitoyl-2-oleoyl-sn-glycero-3-phosphatidylinositol 4,5-bisphosphate (PIP_2_) generated using CHARMM-GUI membrane builder^64^. The initial position of PIP_2_ was at least 15 Å away from the protein surface. The membrane/protein systems were then solvated with TIP3P water and neutralized with 150 mM KCl.

All the MD simulations were performed with NAMD2.12^65^ using CHARMM36m force field for lipid/protein^66^ and a timestep of 2 fs. Long range electrostatic interactions were evaluated with particle mesh Ewald (PME)^67^ and periodic boundary conditions were used throughout the simulations. Non-bonded forces were calculated with a cutoff of 12 Å and switching distance of 10 Å. During the simulation, temperature (T = 310 K) and pressure (P = 1 atm) (NPT ensemble) was maintained by Nosé-Hoover Langevin piston method^68^. During pressure control, the simulation box was allowed to fluctuate in all the dimensions with constant ratio in the x-y (lipid bilayer) plane.

### Immunoblot analysis

At 48 h post transfection, the CHOhm1 cells were washed with 1X PBS, and harvested in ice-cold lysis buffer containing (in mM): 50 Tris, 150 NaCl, 2 EGTA, 1 EDTA, 1% Triton, 0.5% deoxycholic acid, 0.1% SDS (pH 7.4) supplemented with Halt protease inhibitors (Thermo Fisher Scientific) as described^12,45^. After 15 min incubation, the cells in lysis buffer were centrifugated at 14,000 x g for 15 min at 4 °C. The lysates were mixed with SDS sample buffer in 1:5 dilution (in mM): containing 75 Tris, 10% SDS, 50 TCEP, 12.5% glycerol, 0.50 EDTA, 0.50 mg/mL Bromophenol Blue. After heating at 75 °C for 30 min, the samples were run on 12% non-gradient and 4–20% gradient SDS-PAGE gels (Bio-Rad), transferred to a polyvinyl difluoride (PVDF) membrane (Immobilon, Millipore), and analyzed by immunoblotting^12,45^. Briefly, the membranes were blocked in blocking buffer (5% milk, 0.1% Tween-20 in TBS), and incubated with mouse anti-K_v_7.2 (1:200 dilution), rabbit anti-K_v_7.3 (1:500 dilution) or anti-GAPDH antibody (1:1000 dilution) in wash buffer (1% milk, 0.1% Tween-20 in TBS) overnight at 4 °C. After incubating with horse radish peroxidase-conjugated secondary antibodies in wash buffer for 1 hr, the blots were washed, and treated with Pierce ECL or SuperSignal Pico Plus substrate (Thermo Fisher Scientific #32106, #34577). The immunoblot membranes were immediately imaged with the iBright CL1000 imaging system (Thermo Fisher Scientific). ImageJ software (NIH, http://rsb.info.nih.gov/ij) was used to measure background-subtracted immunoblot band intensities of K_v_7.2 and K_v_7.3 (monomers, dimers, multimers) and GAPDH as previously decreased^12,45^. The ratio of K_v_7.2/GAPDH and K_v_7.3/GAPDH from K_v_7.2 WT samples were taken as 100% and the ratio of EE mutant samples were normalized to the ratio of WT samples to obtain % K_v_7.2 WT. Antibodies used in immunoblotting include anti-K_v_7.2 (Neuromab, N26A/23), rabbit anti-K_v_7.2 (Alomone, APC-050), rabbit anti-K_v_7.3 (Alomone, APC-051), anti-GAPDH antibodies (Cell Signaling, 2118), donkey anti-rabbit and anti-mouse HRP secondary antibodies (The Jackson Laboratory, 711-035-152, 715-035-150).

### Immunoprecipitation

HEK293T cells were plated on 100 mm cell culture dishes (BD Biosciences, 2 × 10^6^ cells per dish) and maintained in Minimal Essential Medium containing 10% Fetal Bovine Serum, 2 mM glutamine, 100 U/mL penicillin and 100 U/mL streptomycin at 37 °C and 5% CO_2_. At 24 hr post plating, the cells were transfected with plasmids (total 1.6 μg) containing K_v_7.2 and EYFP-hCaM (1:1 ratio), using FuGENE6 transfection reagent (Promega). For coimmunoprecipitation studies of K_v_7.2 and K_v_7.3, the cells were transfected with K_v_7.2 and K_v_7.3 containing an extracellular hemagglutinin epitope (HA-K_v_7.3) (1:1 ratio). At 48 h post transfection, the cells were washed with ice-cold PBS and lysed in ice-cold immunoprecipitation (IP) buffer containing (in mM): 20 Tris-HCl, 100 NaCl, 2 EDTA, 5 EGTA, 1% Triton X-100 (pH 7.4) supplemented with Halt protease inhibitors (Thermo Fisher Scientific). The lysate containing equal amount of proteins were first precleared with Protein A/G agarose beads (100 μL, Santa Cruz) for 1 hr at 4 °C, and then incubated overnight at 4 °C with Protein A/G-agarose beads (100 μL) and rabbit anti-K_v_7.2 antibody (2 μg). This amount of anti-K_v_7.2 antibody allowed us to immunoprecipitate the equal amount of K_v_7.2 proteins and analyze the effects of mutations on the amount of co-immunoprecipitated EYFP-hCaM and HA-K_v_7.3. After washing with IP buffer, the immunoprecipitates were eluted with SDS sample buffer by incubating at 75 °C for 10–15 min, and analyzed by western blot analysis with mouse anti-GFP (1:500 dilution), mouse anti-K_v_7.2 (1:200 dilution), mouse anti-HA antibodies (1:500 dilution), and rabbit anti-GAPDH antibodies (1:1000 dilution). Antibodies used in coimmunoprecipitation and immunoblotting include anti-K_v_7.2 (Neuromab, N26A/23), rabbit anti-K_v_7.2 (Alomone, APC-050), anti-GFP, anti-HA, anti-GAPDH antibodies (Cell Signaling, 2955, 2367, 2118), rabbit anti-K_v_7.3 (Alomone, APC-051), donkey anti-rabbit and anti-mouse HRP secondary antibodies (The Jackson Laboratory, 711–035–152, 715-035-150).

### Immunocytochemistry

All procedures involving animals were reviewed and approved by the Institutional Animal Care and Use Committee at the University of Illinois Urbana-Champaign and conducted in accordance with the guidelines of the U.S National Institute of Health (NIH). Primary rat dissociated hippocampal cultured neurons prepared from 18-day old embryonic rats were plated on 12 mm glass coverslips (Warner Instruments, 10^5^ cells per coverslip) coated with poly L-lysine (0.1 mg/mL). These neurons were maintained in neurobasal medium supplemented with B27 extract, 200 mM L-glutamine, and 100 U/mL penicillin and streptomycin in a cell culture incubator (37 °C, 5% CO_2_). At 5 days *in vitro* (DIV), neurons were transfected with plasmids (total 0.8 μg) containing K_v_7.3 with an extracellular hemagglutinin epitope (HA-K_v_7.3) and wild-type or mutant K_v_7.2 using lipofectamine LTX as described^12,45^.

Immunostaining for surface HA-K_v_7.3 and total K_v_7.2 subunits were performed at 48 h post transfection as described^12,45^. In brief, neurons were washed once with artificial cerebral spinal fluid (ACSF) solution containing (in mM): 10 HEPES, 150 NaCl, 3 KCl, 2 CaCl_2_, 10 Dextrose (pH 7.4). Neurons were fixed in 4% paraformaldehyde / 4% sucrose in Phosphate buffered saline (PBS) for 8 min, washed with PBS, blocked with 10% normal donkey serum (NDS) in PBS for 1 hr. To label surface HA-K_v_7.3, neurons were incubated with rabbit anti-HA antibody (1:500 dilution) in 3% NDS in PBS overnight at 4 °C without permeabilization, followed by incubation with donkey anti-rabbit Alexa488-conjugated secondary antibodies (1:200-1:300 dilution). To label total K_v_7.2 and AIS marker, neurons were fixed for 15 min, permeabilized with 0.2% Triton X-100 in PBS for 30 min, and incubated with goat anti-K_v_7.2 antibody (1:200 dilution) and rabbit anti-phospho IκBα Ser32 (14D4) antibody (1:500 dilution) or mouse anti-Ankyrin G antibody (1:500) in 3% NDS in PBS at 4 °C overnight. After the PBS wash, the neurons were incubated with donkey anti-goat Alexa594-conjugated secondary antibodies (1:200-1:300 dilution) and anti-rabbit Alexa680-conjugated secondary antibodies (1:200-1:300 dilution) for 2 hr. The coverslips were mounted using Fluorogel anti-fade mounting medium (Electron Microscopy Sciences).

Cell permeable dynamin inhibitory peptide (DIP, Tocris Bioscience Cat. No. 1775) and diluted to 50 μM by artificial cerebrospinal fluid (ACSF). Transfected coverslips were incubated in the DIP or vehicle for 45 minutes before immunostaining. Antibodies used in immunofluorescence staining include anti-HA (Cell Signaling, 3724), anti-K_v_7.2 (Santa-Cruz, sc-7793), anti-ankyrin G (Neuromab, 75–146), anti-phospho IκBα Ser32 (14D4) (Cell Signaling, 2859) and Alexa Fluor secondary antibodies (Invitrogen, A10043, A21206, A10038, A11058).

Fluorescence and phase contrast images of transfected neurons were viewed using a Zeiss Axio Observer inverted microscope High-resolution gray scale images of healthy transfected neurons were acquired using a 20X objective with a Zeiss AxioCam 702 mono Camera and ZEN Blue 2.6 software and saved as 16-bit CZI and TIFF files. To compare the fluorescence intensity of the neurons transfected with different constructs, the images were acquired using the same exposure time within one experiment.

The image analyses were performed from the healthy transfected neurons using ImageJ Software as described^12,45^ and excluded the transfected neurons with broken neurites or soma as well as regions where fasciculation or overlapping processes occurred. The axon was identified as a process that were labeled for the AIS marker 14D4, whereas the dendrites were identified as the processes that were absent for 14D4 in the transfected neurons. ImageJ software was used to trace the all major primary dendrites, the AIS (defined as the first 0–30 μm segment of the axon), and distal axon (defined as the segment between 50 and 80 μm from the beginning of the axon) as 1 pixel-wide line segments, and obtain their mean fluorescent intensities. The perimeter of the neuronal soma was also traced to obtain background-subtracted mean fluorescent intensities of the soma.

### Statistical analyses

All analyses are reported as mean ± SEM. Using Origin 9.1 (Origin Lab), the Student *t* test and one-way ANOVA with post-ANOVA Tukey and Fisher’s multiple comparison tests were performed to identify the statistically significant difference with a priori value (p) < 0.05. The number of separate transfected cells for immunostaining and electrophysiology was reported as the sample size n.

## Supplementary information

Supplementary information

Supplementary information2

## Data Availability

The datasets generated during and/or analyzed during the current study are available from the corresponding author on reasonable request.
