# Supplementary material for: Identifying mutation hotspots reveals pathogenetic mechanisms of KCNQ2 epileptic encephalopathy
Source: Sci Rep. 2020 Mar 16;10:4756. doi: 10.1038/s41598-020-61697-6 (PMC7075958; doi:10.1038/s41598-020-61697-6)
Supplement: Supplementary file 2 — Supplementary information2 [file 41598_2020_61697_MOESM2_ESM.pdf]

SUPPLEMENTARY MATERIAL

TITLE: Identifying mutation hotspots reveals pathogenetic mechanisms of *KCNQ2* epileptic encephalopathy

AUTHORS AND AFFILIATIONS:

Jiaren Zhang[1,8], Eung Chang Kim[1,8], Congcong Chen[1,2,8], Erik Procko[3,4], Shashank Pant[3,5,6], Kin Lam[3,5,7], Jaimin Patel[1], Rebecca Choi[1], Mary Hong[1], Dhruv Joshi[1], Eric Bolton[1], Emad Tajkhorshid[3,5,6], Hee Jung Chung,[1,4,*]

[1]Department of Molecular and Integrative Physiology, [2]Department of Statistics, [3]Department of Biochemistry, [4]Neuroscience Program, [5]NIH Center for Macromolecular Modeling and Bioinformatics, Beckman Institute for Advanced Science and Technology, [6]Center for Biophysics and Quantitative Biology, [7]Department of Physics, University of Illinois at Urbana-Champaign, Urbana, Illinois 61801, USA.

[8]These authors contributed equally.

[*]**CORRESPONDING AUTHOR:** Hee Jung Chung

Department of Molecular and Integrative Physiology,

University of Illinois at Urbana-Champaign,

407 South Goodwin Avenue, 524 Burrill Hall, Urbana, IL 61801, USA.

Telephone number: (217) 244-6839

Email: chunghj@life.illinois.edu; chunghj@illinois.edu

SUPPLEMENTARY FIGURES

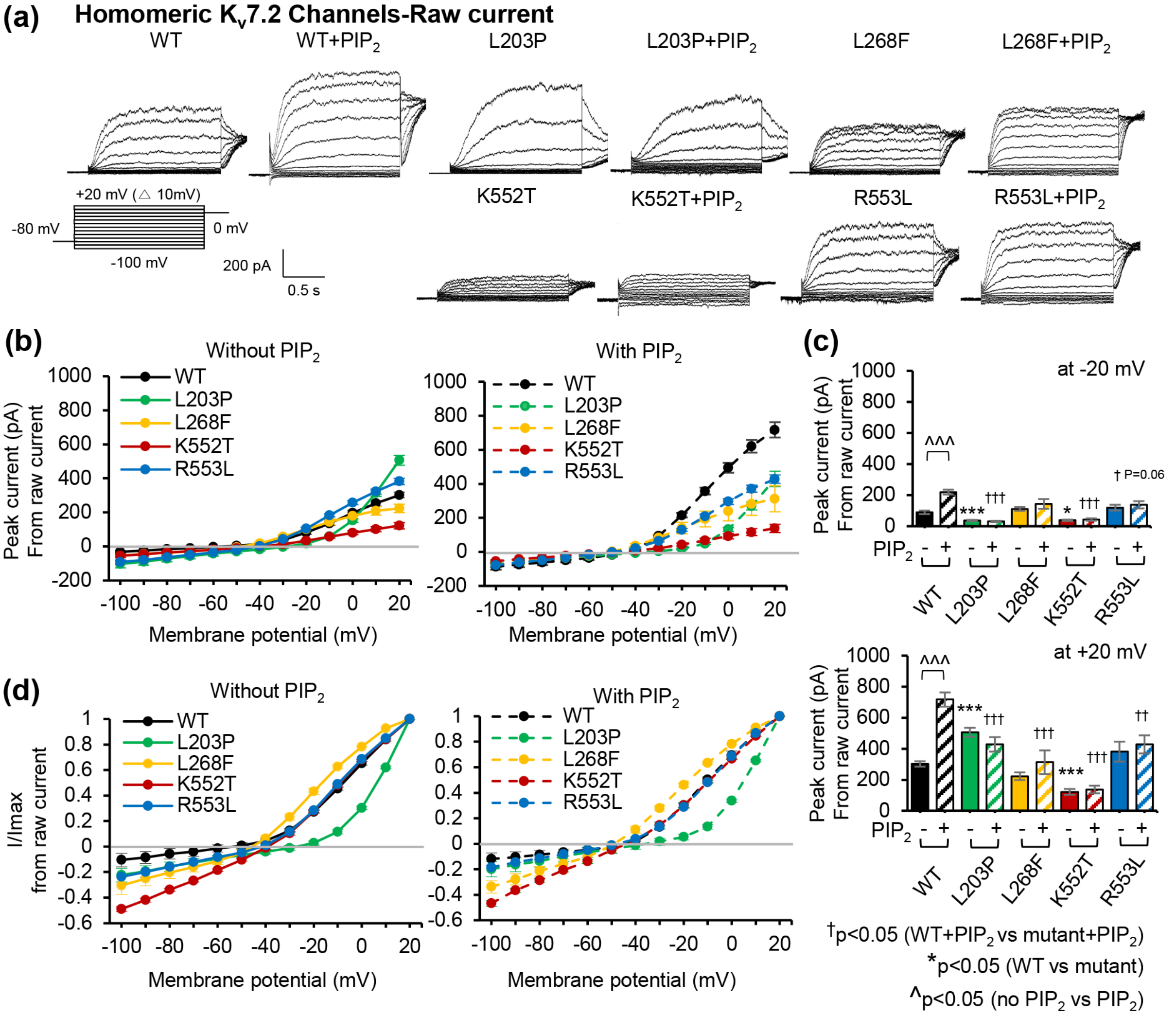

Supplementary Figure S1. The selected EE mutations variably affect voltage-dependent activation of homomeric $K_V7.2$ channels and abolish their current enhancement by application of exogenous diC8-PIP_2. Whole cell patch clamp recordings of macroscopic K^+ currents from GFP-positive CHO hm1 cells expressing $K_V7.2$ WT or EE mutant in the presence or absence of diC8-PIP_2 (100 µM) inclusion in the intracellular pipette. Leak subtracted current traces and data analysis are shown in Figure 3 of the manuscript. **(a)** Representative raw current traces. **(b-c)** Average peak current was quantified from raw currents and represented in **(b)** at all voltage steps and **(c)** at -20 mV (top) and +20 mV (bottom). **(d)** Normalized currents (I/I_{max}) were quantified using raw currents at all voltage steps.

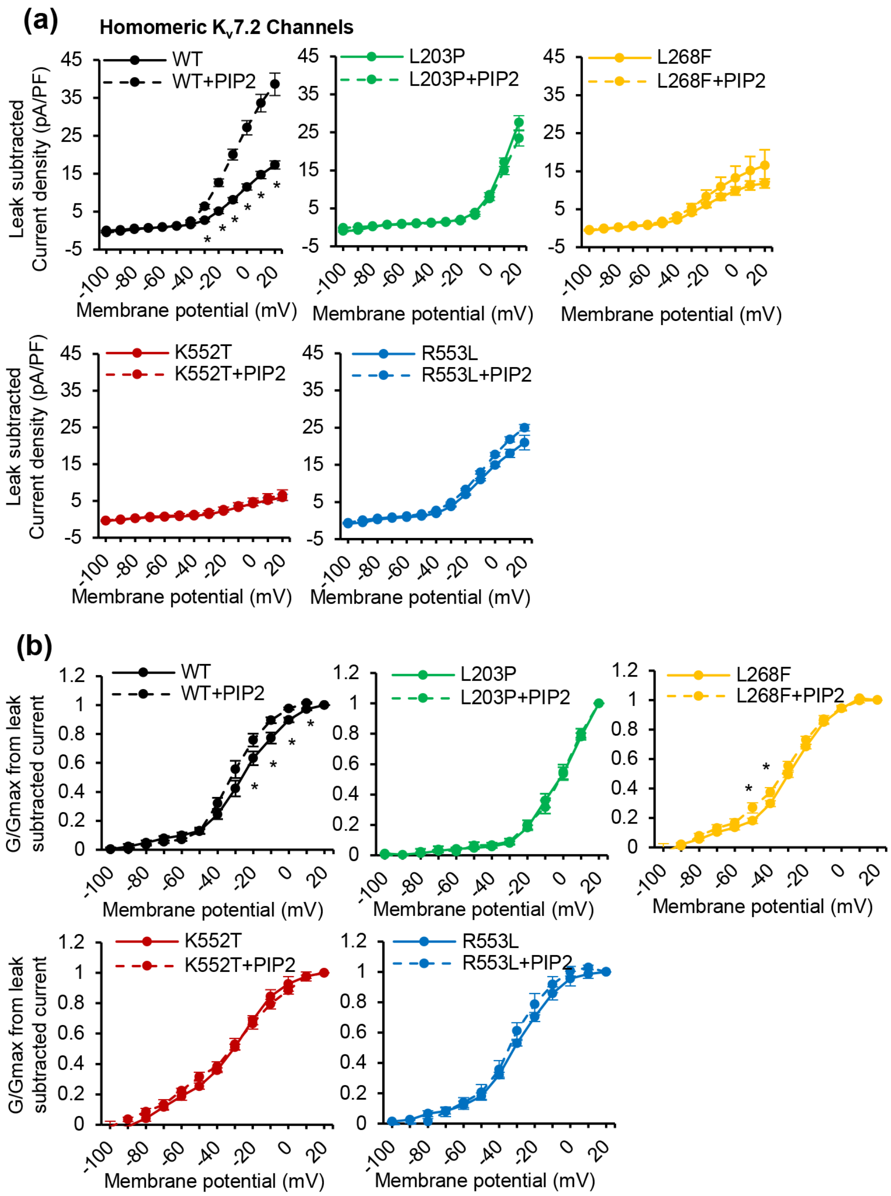

Supplementary Figure S2. All tested EE mutations block current enhancement of homomeric K_v7.2 channels upon application of exogenous diC8-PIP$_2$. Whole cell patch clamp recordings of K^+ currents through homomeric K_v7.2 wild-type (WT) channels and EE mutant channels from GFP-positive CHO hm1 cells in the presence or absence of diC8-PIP$_2$ (100 µM) inclusion in the intracellular pipette (as described in Fig. 3). **(a)** Average current density and **(b)** normalized conductance (G/Gmax) of leak-subtracted currents through homomeric K_v7.2 WT or mutant channels with or without diC8-PIP$_2$ inclusion. *$p<0.05$.

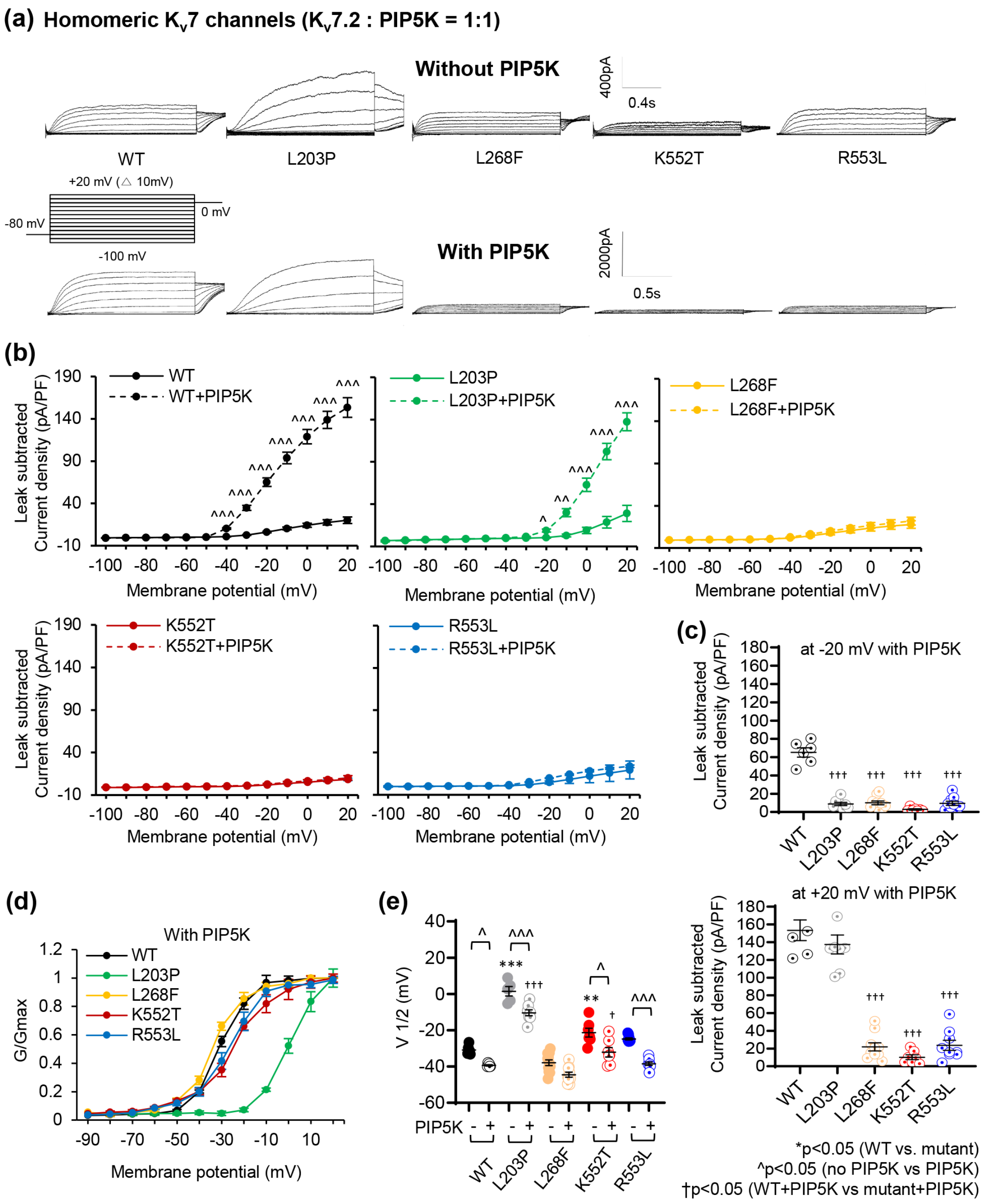

Supplementary Figure S3. L268F, K552T and R553L variants block current enhancement of homomeric K$_V$7.2 channels upon co-expression of PIP5K. Whole cell patch clamp recordings of K$^+$ currents through homomeric K$_V$7.2 wild-type (WT) channels and EE mutant channels from GFP-positive CHOhm1 cells co-transfected PIP5K. Cells were held at -80 mV. Currents were evoked by depolarization for 1.5 s from −100 mV to +20 mV in 10 mV increments, followed by a step to 0 mV for 300 ms. **(a)** Representative recordings of K$_V$7.2 currents after subtraction of leak currents. Leak current was defined as non-voltage-dependent current from GFP-transfected cells. **(b)** Average K$_V$7.2 peak current densities at all voltage steps. **(c-d)** Average K$_V$7.2 peak current densities at -20 mV (top) and + 20 mV (bottom) (†p<0.05, †††p<0.005 based on Fisher's test) **(c)** and Normalized conductance (G/Gmax) of K$_V$7.2 currents

(d) from GFP-positive CHOhm1 cells co-transfected with PIP5K. **(e)** Average half-activation potential $V_{1/2}$ was calculated from normalized conductance G/Gmax. The *p*-values are computed from post-hoc Tukey tests. The number of GFP-positive cells without PIP5K: K_v7.2 WT (n=6), L203P (n=5), L268F (n=11), K552T (n=7), or R553L (n=10). The number of GFP-positive cells that were cotransfected with PIP5K: K_v7.2 WT (n=6), L203P (n=8), L268F (n=10), K552T (n=7), or R553L (n=9). Data shown represent the Ave ± SEM.

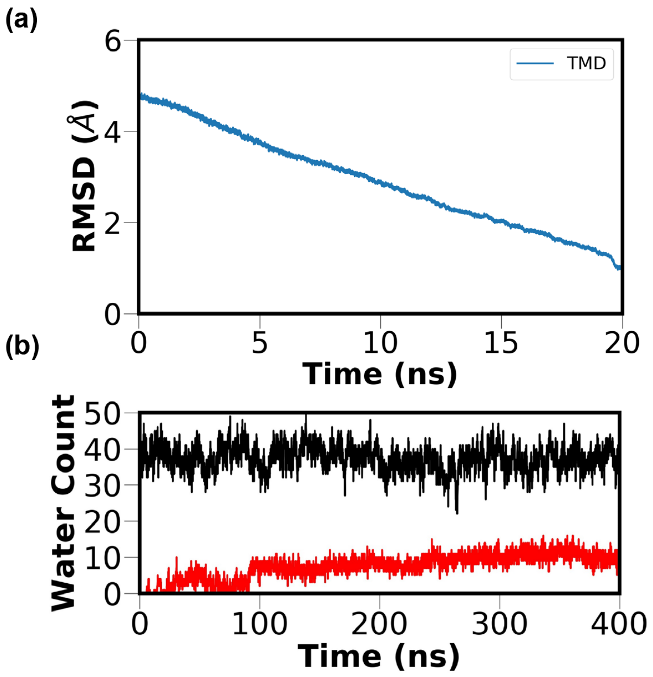

Supplementary Figure S4. The completion of non-equilibrium simulation and the stability of the modeled open-conformation K_v7.2 channel structure are tested and confirmed. (a) The success of our targeted MD simulations was gauged by measuring the RMSD (root-mean-square deviation) of S4-S5 and S6 helices with respect to the target open state. Our target open state is the published structure of chimeric K_v1.2/K_v2.1 channel in the open-state conformation. In 20 ns, RMSD reaches the level of <1 Å, suggesting that our targeted MD simulation was successful. **(b)** The water count in the pore helix and selectivity filter of open (black) and closed (red) states of our model. The water count is defined by the number of water molecules within the pore-helix and selectivity filter region of K_v7.2. The stable water count of the open-state conformation suggests that our model is relatively stable during the simulated time scale.

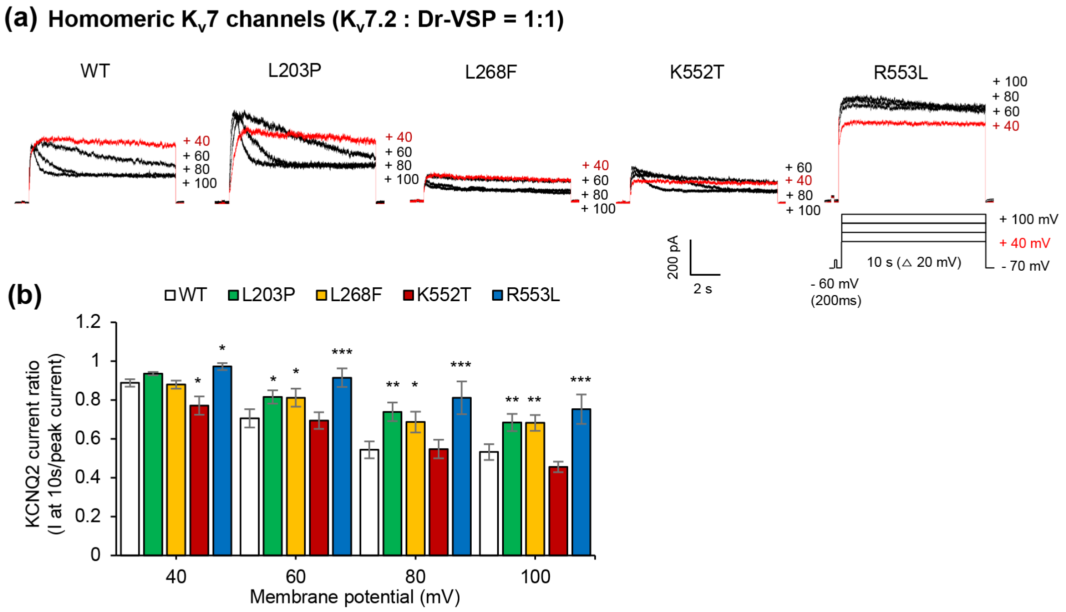

Supplementary Figure S5. The effects of Dr-VSP activation on currents through $K_v7.2$ WT and EE mutant channels. Whole cell voltage clamp recordings of macroscopic K^+ currents in CHO hm1 cells transfected with *danio rerio* voltage-sensitive phosphatase (Dr-VSP) and $K_v7.2$ WT or EE mutants. CHO cells were held at - 70 mV and a brief voltage step to –60 mV was applied to calculate the linear leak. Currents were evoked by depolarization from −20 mV to +100 mV in 20 mV increments. Duration of depolarization was 10 s to allow depolarization-induced activation of Dr-VSP and PIP_2 depletion. There were 2 min inter-step intervals to allow PIP_2 regeneration. **(a)** Representative current traces of $K_v7.2$ WT and EE mutants at voltage steps from +40 mV to +100 mV (instead of all voltage steps) were shown for clarity. **(b)** The average ratio of $K_v7.2$ current at 10 s over peak $K_v7.2$ current at voltage steps from +40 mV to +100 mV. The depolarization step at voltages more positive than +40 mV decreased peak currents of $K_v7.2$ channels which reached maximum decay at +100 mV. The K552T mutation increased Dr-VSP-mediated decay of $K_v7.2$ current at +40 mV, whereas the L203P, L268F, and R553L mutations decreased Dr-VSP-mediated decay of $K_v7.2$ currents from +40 to +100 mV. *$p<0.05$ based on one-way ANOVA Fisher's test. The number of Dr-VSP-cotransfected cells: Kv7.2 WT (n=10), L203P (n=14), L268F (n=12), K552T (n=13), or R553L (n=8). Data shown represent the Ave ± SEM.

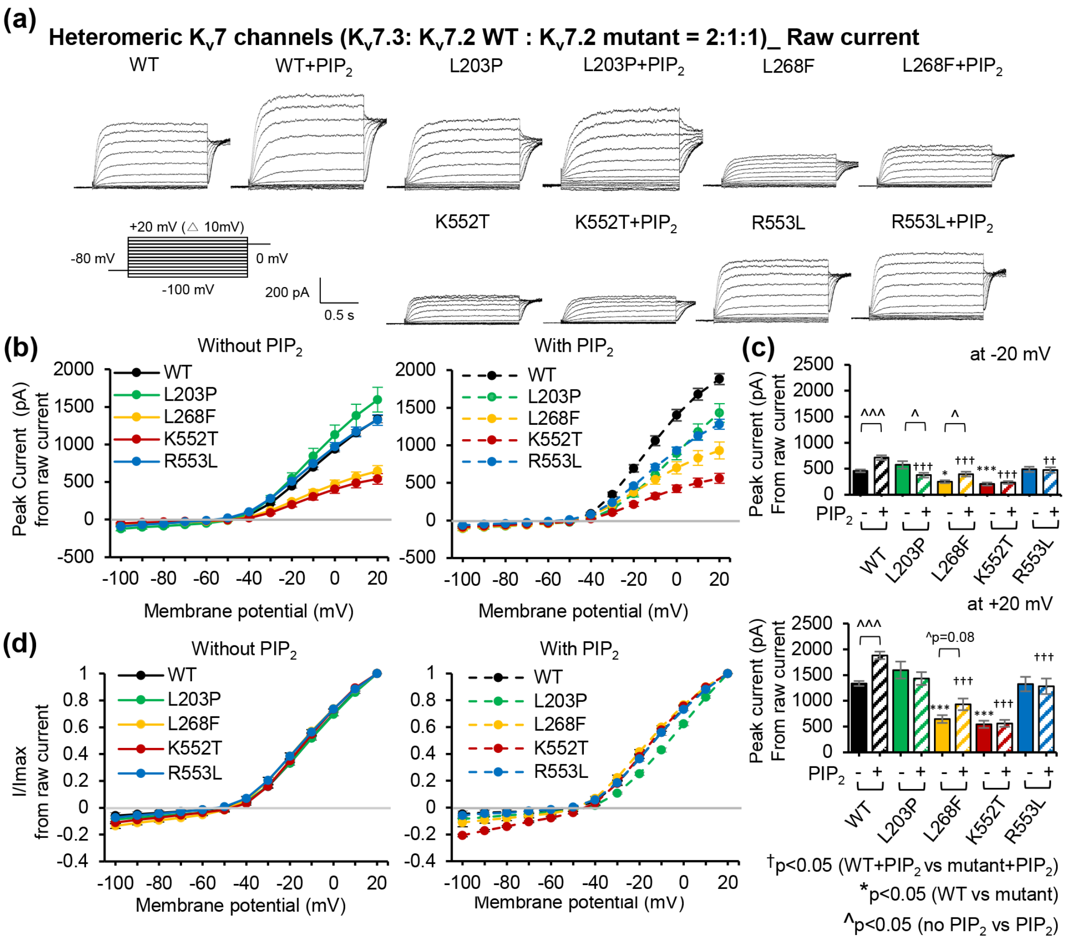

Supplementary Figure S6. All tested EE mutations abolish current enhancement of heteromeric K_V7 channels by application of exogenous diC8-PIP_2 but only L268F and K552T mutations reduced their current density. Whole cell patch clamp recordings of macroscopic K^+ currents from GFP-positive CHO hm1 cells expressing $K_V7.3$ and $K_V7.2$ WT (1:1 ratio) or $K_V7.3$, $K_V7.2$ WT, $K_V7.2$ EE mutant (2:1:1 ratio). Leak subtracted current traces and data analysis are shown in Figure 5 of the manuscript. **(a)** Representative raw current traces. **(b-c)** Average peak current was quantified from raw currents and represented in **(b)** at all voltage steps and **(c)** -20 mV (top) and +20 mV (bottom). **(d)** Normalized currents (I/I_{max}) were quantified using raw currents at all voltage steps.

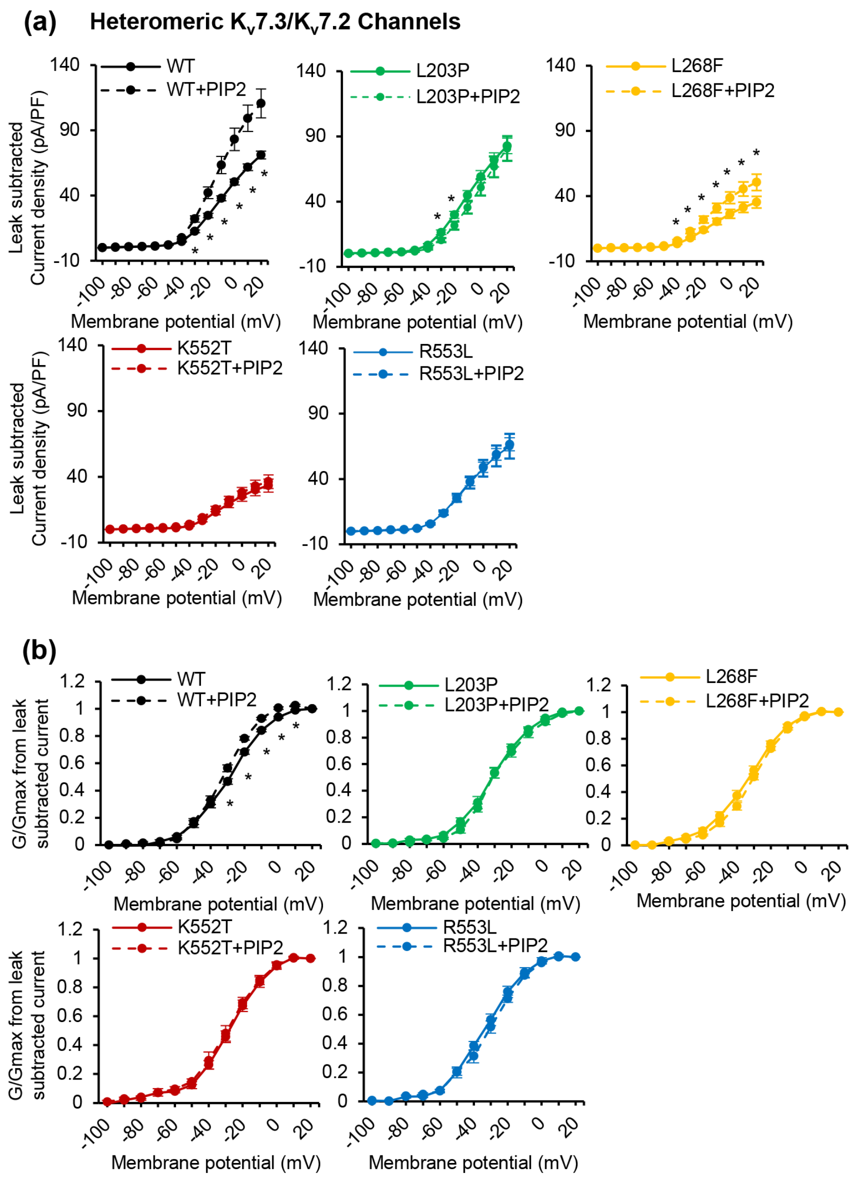

Supplementary Figure S7. All tested EE mutations block current enhancement of heteromeric K_v7 channels upon application of exogenous diC8-PIP$_2$. Whole cell patch clamp recordings of K^+ currents from GFP-positive CHO hm1 cells cotransfected with K_v7.3 and K_v7.2 WT (1:1 ratio) or K_v7.3, K_v7.2 WT, K_v7.2 mutant (2:1:1 ratio) in the presence or absence of diC8-PIP$_2$ (100 μM) inclusion in the intracellular pipette (as described in Fig. 5). **(a)** Average current density and **(b)** normalized conductance (G/Gmax) of leak-subtracted currents through heteromeric K_v7 channels with or without diC8-PIP$_2$ inclusion. $^*p<0.05$.

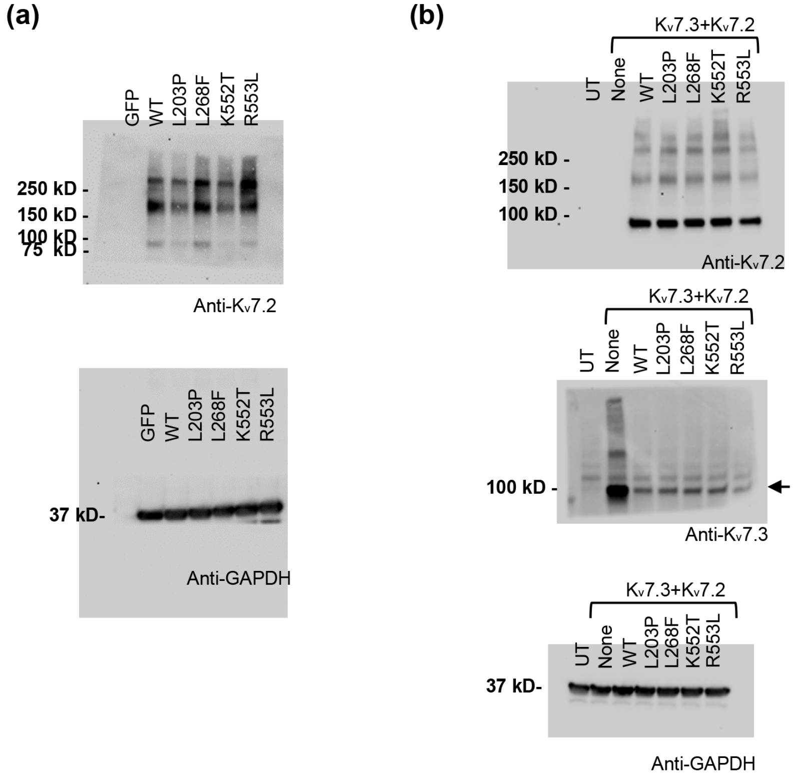

Supplementary Figure S8. Original blot of western blot images shown in Fig. 3, 5. (a) Original image of the blot shown in Fig. 3c. **(b)** Original image of the bot shown in Fig. 5f.

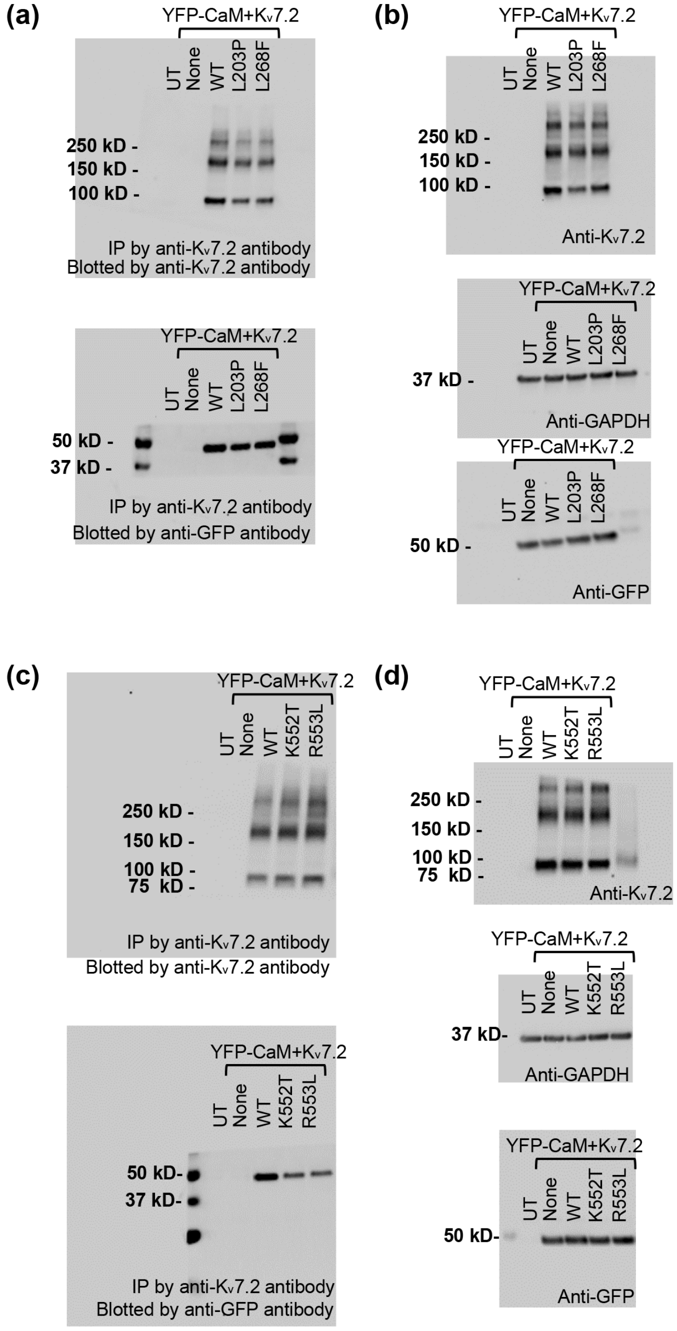

Supplementary Figure S9. Original blot of western blot images shown in Fig. 6a. (a, c) IP (immunoprecipitated). (b, d) total lysate. Anti-GFP antibody is used to visualize YFP-CaM.

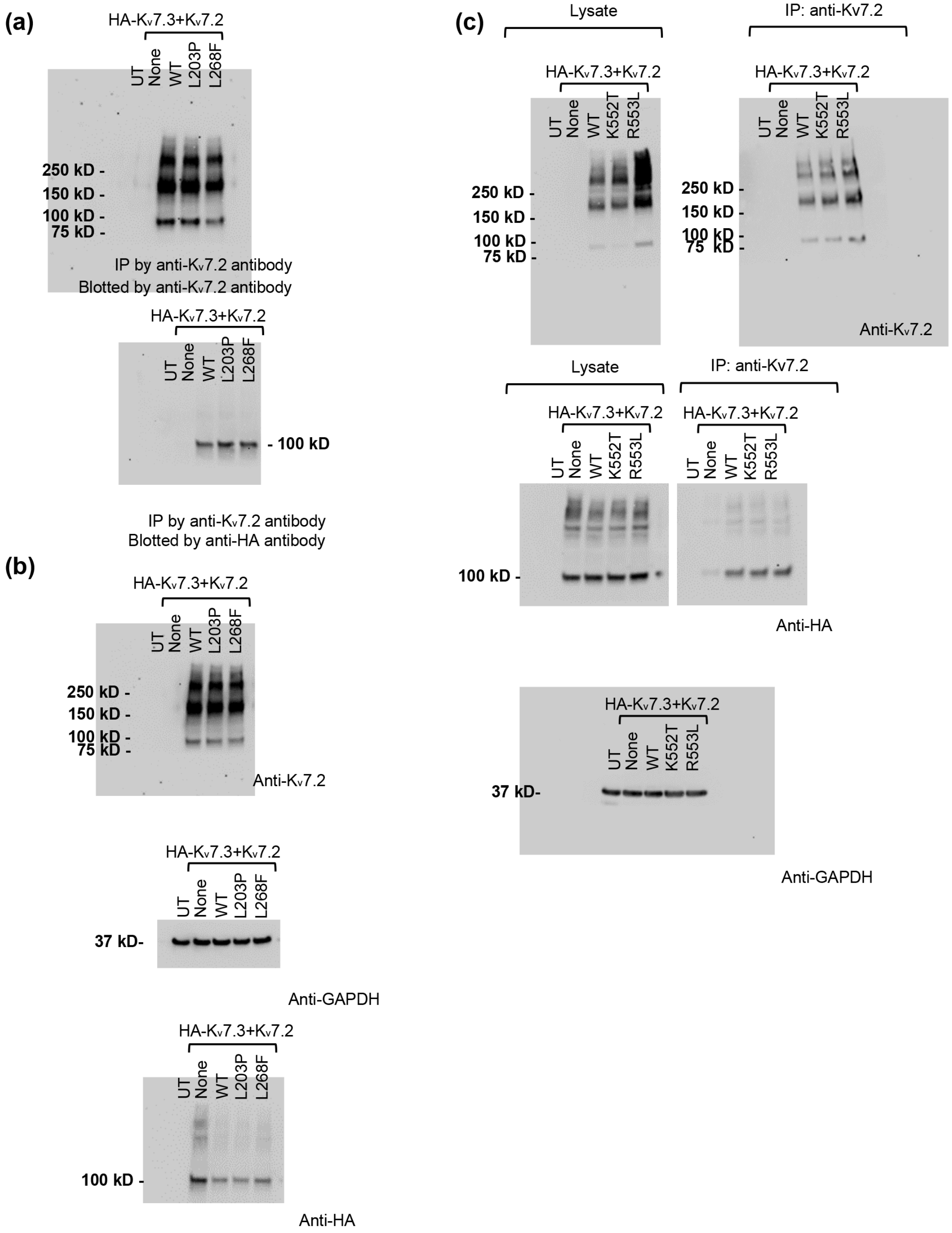

Supplementary Figure S10. Original blot of western blot images shown in Fig. 6c. (a, b) Uncropped images of coIP studying the interaction between HA-K_v7.3 and K_v7.2 WT, L203P or L268F mutants. **(c)** Uncropped images of coIP studying the interaction between HA-K_v7.3 and K_v7.2 WT, K552T or R553L mutants. Anti-HA antibody was used to visualize HA-K_v7.3.

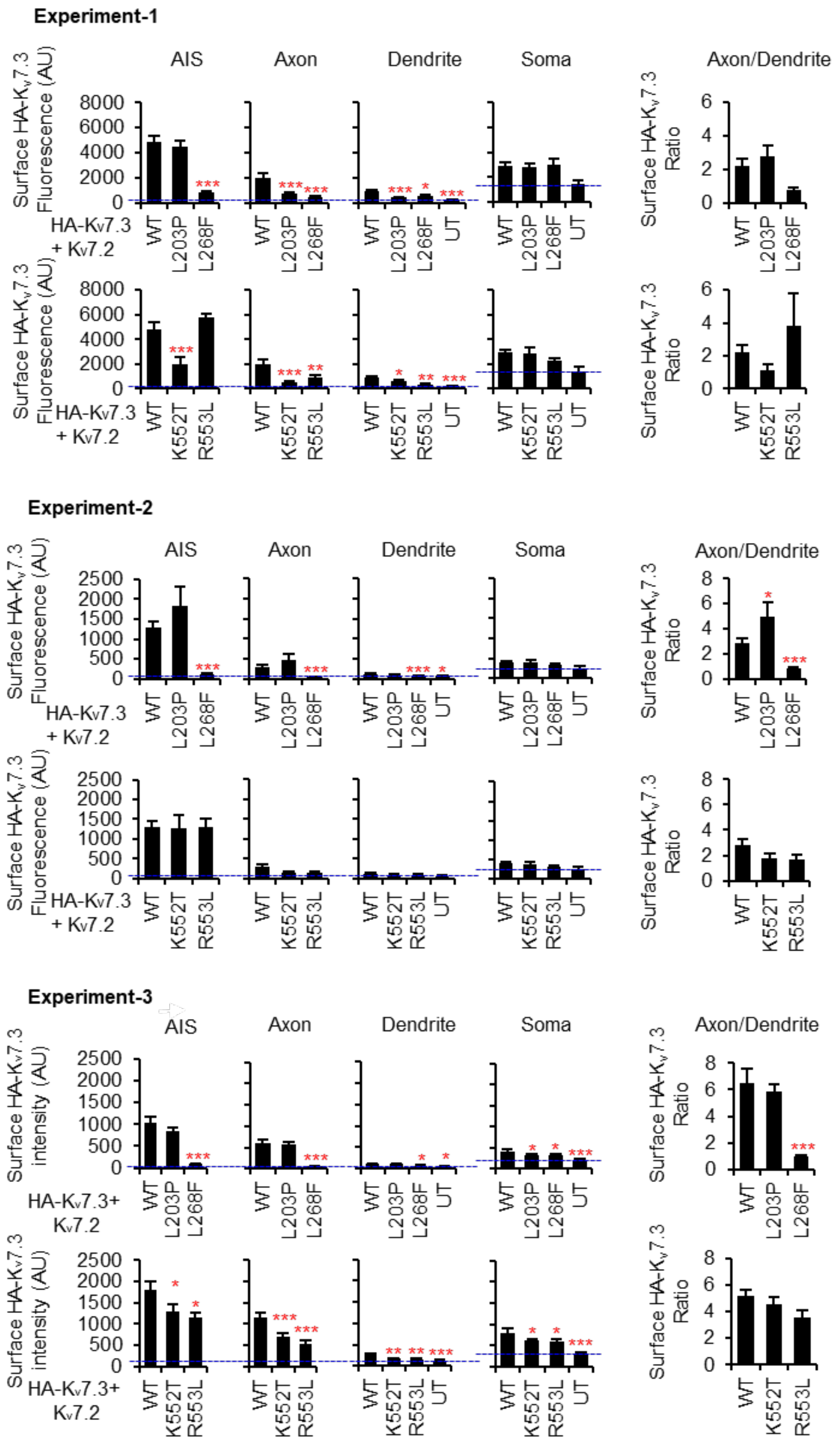

Supplementary Figure S11. Tested EE mutations variably affected surface expression of heteromeric HA-K$_V$7.3/K$_V$7.2 channels. Immunostaining of surface HA-Kv7.3 in healthy hippocampal neurons cotransfected with K$_V$7.2 WT or K$_V$7.2 with EE mutations L203P, L268F, K552T, and R553L. Figures represent data from three separate experiments and are combined and shown in Fig. 7. Data represent the Ave ± SEM (*p<0.05, **p<0.01, ***p<0.005 based on one-way ANOVA Fisher's test.)

SUPPLEMENTARY VIDEO

Supplementary Video 1. All-atom MD simulations of the $K_v7.2$ (open conformation) in PIP_2 containing lipid bilayers. Our simulations started with the unbound form of PIP_2. During 500ns of MD simulations, we have captured the binding of PIP_2 to K552-R553-K554 on helix B. Helices A, B and C of $K_v7.2$ are shown in cartoon representation, and the rest of the protein is shown in surface representation. PIP_2 lipid is shown in vdW (van der Waals) representation (carbon atoms are shown in yellow, oxygen atoms in red and phosphorous atoms in tan color). PIP_2 interaction site (K552-R553-K554) on helix B is highlighted in licorice representation.

SUPPLEMENTARY TABLES

Supplementary Table S1. Epilepsy mutations in $K_v7.2$ which were identified by 12/31/2017.

Protein	Mutation	Location	cDNA change	Mutation type	Primary Disease	Reference
$K_v7.2$	*M1T	N-terminal	c.2T>C	Non-initiation	Mild/BFNE	Grinton BE et al., 2015 (PMID 25982755)
$K_v7.2$	*M1V	N-terminal	c.1A>G	Non-initiation	Uncertain severity	Grinton BE et al., 2015 (PMID 25982755); Milh M et al., 2015 (PMID 25959266); Richards MC et al., 2004 (PMID 14985406)
$K_v7.2$	S105del	S1	c.314-316CCTdel	Single a.a. deletion	Mild/BFNE	Claes LR et al., 2004 (PMID 15596769)
$K_v7.2$	L107F	S1	c.319C>T	Missense	Mild/BFNE	Hortiguela M et al., 2017 (PMID 27535030)
$K_v7.2$	T114A	S1-S2 linker	c.340A>G	Missense	Mild/BFNE	Grinton BE et al., 2015 (PMID 25982755)
$K_v7.2$	T114I	S1-S2 linker	c.341C>T	Missense	Severe/EE	Saitsu S et al., 2012 (PMID 22926866)
$K_v7.2$	K116del	S1-S2 linker	c.346-348AAGdel	Single a.a. deletion	Mild/BFNE	Neubauer BA et al., 2008 (PMID 18625963)
$K_v7.2$	E119G	S1-S2 linker	c.356A>G	Missense	Mild/BFNE	Wuttke TV et al., 2008 (PMID 18006581)
$K_v7.2$	S122L	S1-S2 linker	c.365C>T	Missense	Mild/BFNE	Hunter J et al., 2006 (PMID 16916607)
$K_v7.2$	E130K	S2	c.388G>A	Missense	Severe/EE	Hortiguela M et al., 2017 (PMID 27535030)
$K_v7.2$	R144Q	S2-S3 linker	c.431G>A	Missense	Severe/EE	Miceli F et al., 2015 (PMID 25740509); Allen AS et al., 2013 (PMID 23934111)
$K_v7.2$	Y154D	S2-S3 linker	c.460T>G	Missense	Mild/BFNE	Zara F et al., 2013 (PMID 23360469)
$K_v7.2$	G159R	S2-S3 linker	c.475G>A	Missense	Mild/BFNE	Grinton BE et al., 2015 (PMID 25982755)
$K_v7.2$	G159E	S2-S3 linker	c.476G>A	Missense	Mild/BFNE	Zara F et al., 2013 (PMID 23360469)
$K_v7.2$	V175L	S3	c.523G>T; c.523G>C	Missense	Severe/EE	Milh M et al., 2013 (PMID 23692823); Devaux J et al., 2016 (PMID 27030113); Samanta D et al, 2015 (PMID 25092550)
$K_v7.2$	S187Y	S3	c.560C>A	Missense	Severe/EE	Gokben S et al., 2017 (PMID 27734276)
$K_v7.2$	G189V	S3-S4 linker	c.566G>T	Missense	Severe/EE	Milh et al., 2013 (PMID 236928230); Milh M et al., 2015 (PMID 25959266)
$K_v7.2$	N190S	S3-S4 linker	c.569A>G	Missense	Severe/EE	Millichap et al., 2016 (PMID 27602407)
$K_v7.2$	S195P	S3-S4 linker	c.583T>C	Missense	Severe/EE	Weckhuysen S et al., 2013 (PMID 24107868)
$K_v7.2$	A196V	S4	c.587C>T	Missense	Uncertain severity	Zara F et al., 2013 (PMID 23360469); Soldovieri MV et al., 2014 (PMID 24375629); Carvill GL et al., 2013 (PMID 23708187); Soldovieri MV et al., (PMID 18698150)
$K_v7.2$	R198Q	S4	c.593G>A	Missense	Severe/EE	Millichap et al., 2017 (PMID 27861786)
$K_v7.2$	R201C	S4	c.601C>T	Missense	Severe/EE	Trump et al., 2016 (PMID 26993267); Millichap et al., 2016 (PMID 27602407); Weckhuysen S et al., 2013 (PMID 24107868); Hortiguela et al., 2017 (PMID 27535030); Miceli F et al., 2015 (PMID 25740509)
$K_v7.2$	R201H	S4	c.602G>A	Missense	Severe/EE	Carvill GL et al., 2013 (PMID 23708187); Miceli F et al., 2015 (PMID 25740509); Pisano T et al., 2015 (PMID 25880994)
$K_v7.2$	L203P	S4	c.608T>C	Missense	Severe/EE	Milh M et al., 2013 (PMID 23692823)
$K_v7.2$	I205V	S4	c.613A>G	Missense	Severe/EE	Pisano T et al., 2015 (PMID 25880994); Niday Z et al., 2017 (PMID 28100740); Weckhuysen S et al., 2012 (PMID 22275249); Orhan G et al., 2014 (PMID 24318194)
$K_v7.2$	R207Q	S4	c.620G>A	Missense	Uncertain severity	Wuttke TV et al., 2007 (PMID 17872363); Milh M et al., 2015 (PMID 25959266)

$K_v7.2$	R207W	S4	c.619C>T	Missense	Uncertain severity	Wuttke TV et al., 2007 (PMID 17872363); Milh M et al., 2015 (PMID 25959266); Dedek et al., 2001 (PMID 11572947); Steinlein OK et al., 2007 (PMID 17129708); Sands T et al., 2016 (PMID 27888506); Soldovieri MV et al., 2013 (PMID 24375629)
$K_v7.2$	M208V	S4	c.622A>G	Missense	Mild/BFNE	Singh NA et al., 2003 (PMID 14534157)
$K_v7.2$	R210H	S4	c.629G>A	Missense	Severe/EE	Reid et al., 2016 (PMID 26446091); Numis AL et al., 2014 (PMID 24371303); Pisano T et al., 2015 (PMID 25880994); Weckhuysen et al., 2013 (PMID 24107868)
$K_v7.2$	R210C	S4	c.628C>T	Missense	Severe/EE	Mercimek-Mahmutoglu S et al., 2015 (PMID 25818041)
$K_v7.2$	D212G	S4	c.635A>G	Missense	Mild/BFNE	Miceli F et al., 2009 (PMID 19344764)
$K_v7.2$	R213W	S4	c.637C>T	Missense	Severe/EE	Zhang Q et al., 2017 (PMID 27779742); Trump et al., 2016 (PMID 26993267); Milh M et al., 2015 (PMID 25959266)
$K_v7.2$	R213Q	S4	c.638G>A	Missense	Severe/EE	Trump et al., 2016 (PMID 26993267); Grinton BE et al., 2015 (PMID 25982755); Pisano T et al., 2015 (PMID 25880994); Weckhuysen S et al., 2012 (PMID 22275249); Orhan G et al., 2014 (PMID 24318194)
$K_v7.2$	R214W	S4	c.640C>T	Missense	Mild/BFNE	Miraglia del Giudice et al., 2000 (PMID 11175290); Castaldo P et al., 2002 (PMID 11784811)
$K_v7.2$	G215R	S4	c.643G>A	Missense	Severe/EE	Meurs-van der Schoor et al., 2014 (PMID 25566516)
$K_v7.2$	T217N	S4	c.650C>A	Missense	Severe/EE	Kato M et al., 2013 (PMID 23621294); Surti TS et al., 2005 (PMID 16319223)
$K_v7.2$	T217A	S4	c.649A>G	Missense	Mild/BFNE	Zara F et al., 2013 (PMID 23360469)
$K_v7.2$	H228Q	S4-S5 linker	c.684C>A	Missense	Mild/BFNE	Singh NA et al., 2003 (PMID 14534157)
$K_v7.2$	T234P	S5	c.700A>C	Missense	Severe/EE	Mercimek-Mahmutoglu S et al., 2015 (PMID 25818041)
$K_v7.2$	Y237F	S5	c.710A>T	Missense	Severe/EE	Zhang Q et al., 2017 (PMID 27779742)
$K_v7.2$	G239R	S5	c.715G>C	Missense	Severe/EE	Milh M et al., 2015 (PMID 25959266); Milh M et al., 2013 (PMID 23692823)
$K_v7.2$	L243F	S5	c.727C>T	Missense	Mild/BFNE	Singh NA et al., 2003 (PMID 14534157); Seebohm G et al., 2001 (PMID 11278406)
$K_v7.2$	A246P	S5	c.736G>C	Missense	Severe/EE	Zhang Q et al., 2017 (PMID 27779742)
$K_v7.2$	S247W	S5	c.740C>G	Missense	Severe/EE	Steinlein OK et al., 2007 (PMID 17129708); Kato M et al., 2013 (PMID 23621294); Dedek et al., 2003 (PMID 11572947)
$K_v7.2$	V250G	S5	c.749T>G	Missense	Mild/BFNE	Moulard B et al., 2001 (PMID 11690625)
$K_v7.2$	V250L	S5	c.748G>T	Missense	Severe/EE	Zhang Q et al., 2017 (PMID 27779742)
$K_v7.2$	A253T	Pore	c.757G>A	Missense	Severe/EE	Milh M et al., 2015 (PMID 25959266);
$K_v7.2$	G256W	Pore	c.766G>T	Missense	Severe/EE	Millichap et al.,2016 (PMID 27602407)
$K_v7.2$	N258S	Pore	c.773A>G	Missense	Mild/BFNE	Maljevic S et al., 2011 (PMID 21913284)
$K_v7.2$	D259Y	Pore	c.775G>T	Missense	Mild/BFNE	Hortiguela M et al., 2017 (PMID 27535030)
$K_v7.2$	A265T	Pore	c.793G>A	Missense	Severe/EE	Zhang Q et al., 2017 (PMID 27779742); Millichap et al.,2016 (PMID 27602407); Hortiguela M et al., 2017 (PMID 27535030); Milh M et al., 2013 (PMID 23692823); Weckhuysen S et al., 2013 (PMID 24107868)
$K_v7.2$	A265V	Pore	c.794C>T	Missense	Severe/EE	Saitsu S et al., 2012 (PMID 22926866); Milh M et al., 2015 (PMID 25959266)
$K_v7.2$	A265P	Pore	c.793G>C	Missense	Severe/EE	Weckhuysen S et al., 2012 (PMID 22275249); Orhan et al., 2014 (PMID 24318194)
$K_v7.2$	D266E	Pore	c.798T>A	Missense	Severe/EE	Parrini et al., 2017 (PMID 27864847)
$K_v7.2$	L268P	Pore	c.803T>C	Missense	Severe/EE	Hortiguela M et al., 2017 (PMID 27535030)
$K_v7.2$	L268F	Pore	c.802C>T	Missense	Severe/EE	Pisano T et al., 2015 (PMID 25880994)
$K_v7.2$	W269L	Pore	NR	Missense	Severe/EE	Millichap et al.,2016 (PMID 27602407)
$K_v7.2$	W269T	Pore	c.807G>A	Missense	Mild/BFNE	Sands T et al., 2016 (PMID 27888506)
$K_v7.2$	G271V	Pore	c.812G>T	Missense	Mild/BFNE	Zhou X et al., 2006 (PMID 16691402); Wang J et al., 2015 (PMID 25960349); Zhou X et al., 2016 (PMID 28078031)

K_v7.2	T274M	Pore	c.821C>T	Missense	Severe/EE	Zhang Q et al., 2017 (PMID 27779742); Millichap et al., 2016 (PMID 27602407); Hortiguela M et al., 2017 (PMID 27535030); Milh M et al., 2015 (PMID 25959266); Milh M et al., 2013 (PMID 23692823); Weckhuysen S et al., 2013 (PMID 24107868); Orhan et al., 2014 (PMID 24318194)
K_v7.2	T276I	Pore	c.827C>T	Missense	Severe/EE	Martin HC et al., 2014 (PMID 24463883)
K_v7.2	G279S	Pore	c.835G>A	Missense	Uncertain severity	Gokben S et al., 2017 (PMID 27734276); Schroeder et al., 1998 (PMID 9872318); Peters HC et al., 2005 (PMID 15608631)
K_v7.2	G279C	Pore	c.835G>T	Missense	Severe/EE	Milh M et al., 2015 (PMID 25959266)
K_v7.2	Y280H	Pore	c.838T>C	Missense	Severe/EE	Zhang Q et al., 2017 (PMID 27779742)
K_v7.2	G281W	Pore	c.841G>T	Missense	Severe/EE	Pisano T et al., 2015 (PMID 25880994)
K_v7.2	G281R	Pore	c.841G>A	Missense	Severe/EE	Weckhuysen S et al., 2013 (PMID 24107868)
K_v7.2	Y284C	Pore	c.851A>G	Missense	Mild/BFNE	Tomonoh Y et al., 2014 (PMID 24586341); Ihara et al., 2016 (PMID 26910900); Schroeder BC et al., 1998 (PMID 9872318); Schwake M et al., 2000 (PMID 10788442); Chung HJ et al.,2006 (PMID 16735477)
K_v7.2	Y284H	Pore	c.850T>C	Missense	Severe/EE	Zhang Q et al., 2017 (PMID 27779742)
K_v7.2	Y284D	Pore	c.850T>G	Missense	Severe/EE	Hortiguela M et al., 2017 (PMID 27535030)
K_v7.2	P285H	Pore	c.854C>A	Missense	Severe/EE	Kato M et al., 2013 (PMID 23621294)
K_v7.2	T287N	Pore	c.860C>A	Missense	Severe/EE	Milh M et al., 2015 (PMID 25959266); Milh M et al., 2013 (PMID 23692823)
K_v7.2	G290S	Pore	c.868G>A	Missense	Severe/EE	Milh M et al., 2015 (PMID 25959266); Milh M et al., 2013 (PMID 23692823); Zhang Q et al., 2017 (PMID 27779742)
K_v7.2	G290D	Pore	c.869G>A	Missense	Severe/EE	Orhan et al., 2014 (PMID 24318194); Weckhuysen S et al., 2012 (PMID 22275249)
K_v7.2	R291S	Pore	c.873G>T	Missense	Severe/EE	Zhang Q et al., 2017 (PMID 27779742); Parrini et al., 2017 (PMID 27864847)
K_v7.2	R291G	Pore	c.871A>G	Missense	Severe/EE	Zhang Q et al., 2017 (PMID 27779742)
K_v7.2	A294V	S6	c.881C>T	Missense	Severe/EE	Parrini et al., 2017 (PMID 27864847); Millichap et al.,2016 (PMID 27602407); Hortiguela M et al., 2017 (PMID 27535030); Milh M et al., 2015 (PMID 25959266); Milh M et al., 2013 (PMID 23692823); Pisano T et al., 2015 (PMID 25880994); Abidi et al., 2015 (PMID 26007637)
K_v7.2	A294G	S6	c.881C>G	Missense	Mild/BFNE	Abidi et al., 2015 (PMID 26007637); Steinlein OK et al., 2007 (PMID 17129708)
K_v7.2	T296P	S6	c.886A>C	Missense	Severe/EE	Milh M et al., 2013 (PMID 23692823)
K_v7.2	G301S	S6	c.901G>A	Missense	Severe/EE	Parrini et al., 2017 (PMID 27864847); Milh M et al., 2015 (PMID 25959266)
K_v7.2	F304S	S6	c.911T>C	Missense	Severe/EE	Milh M et al., 2015 (PMID 25959266); Milh M et al., 2013 (PMID 23692823)
K_v7.2	F304del	S6	c.910_912del TTC or TTT	Single a.a. deletion	Mild/BFNE	Ishii A et al., 2009 (PMID 18640800); Ishii A et al., 2012 (PMID 22884718)
K_v7.2	F305del	S6	c.913_915delTTC	Single a.a. deletion	Severe/EE	Štěrbová K et. al., 2018 (PMID 29720203)
K_v7.2	F305L	S6	c.913T>C; c.915C>A	Missense	Severe/EE	Zhang Q et al., 2017 (PMID 27779742); Weckhuysen S et al., 2013 (PMID 24107868)
K_v7.2	A306T	S6	c.916G>A; c.917C>T	Missense	Mild/BFNE	Tomonoh Y et al., 2014 (PMID 24586341); Ihara et al., 2016 (PMID 26910900); Dimassi et al., 2016 (PMID 26138355); Otto JF et al., 2009 (PMID 19453707); Schroeder BC et al., 1998 (PMID 9872318); Schwake M et al., 2000 (PMID 10788442); Chung HJ et al.,2006 (PMID 16735477); Soldovieri MV et al., 2014 (PMID 24375629); Singh NA et al., 2008 (PMID 18483067)
K_v7.2	A306V	S6	c.917C>T	Missense	Severe/EE	Hortiguela M et al., 2017 (PMID 27535030); Milh M et al., 2015 (PMID 25959266)
K_v7.2	A309V	S6	c.926C>T	Missense	Severe/EE	Milh M et al., 2015 (PMID 25959266); Milh M et al., 2013 (PMID 23692823)
K_v7.2	G315R	pre-helix A	c.943G>C	Missense	Severe/EE	Hortiguela M et al., 2017 (PMID 27535030); Weckhuysen S et al., 2013 (PMID 24107868)

$K_v7.2$	R325G	Helix A	c.973A>G	Missense	Severe/EE	Pisano T et al., 2015 (PMID 25880994); Soldovieri et al., 2016 (PMID 27905566); Weckhuysen S et al., 2013 (PMID 24107868)
$K_v7.2$	R333W	Helix A	c.997C>T	Missense	Severe/EE	Steinlein OK et al., 2007 (PMID 17129708); Schmitt et al., 2005 (PMID 16039833); Milh M et al., 2013 (PMID 23692823); Kim EC et al., 2018 (PMID 30008368)
$K_v7.2$	R333Q	Helix A	c.998G>A	Missense	Mild/BFNE	Singh NA et al., 2003 (PMID 14534157)
$K_v7.2$	A337T	Helix A	NR	Missense	Severe/EE	Millichap et al.,2016 (PMID 27602407)
$K_v7.2$	A337G	Helix A	c.1010C>G	Missense	Severe/EE	Saitsu S et al., 2012 (PMID 22926866)
$K_v7.2$	L339R	Helix A	c.1016T>G	Missense	Mild/BFNE	Moulard B et al., 2001 (PMID 11690625). Cavaretta JP et al., 2014 (PMID 25077630)
$K_v7.2$	W344R	Helix A	c.1030T>C	Missense	Mild/BFNE	Soldovieri MV et al., 2014 (PMID 24375629)
$K_v7.2$	L351F	Helix A-B linker	c.1051C>T	Missense	Mild/BFNE	Soldovieri MV et al., 2014 (PMID 24375629)
$K_v7.2$	L351V	Helix A-B linker	c.1051C>G	Missense	Mild/BFNE	Soldovieri MV et al., 2013 (PMID 24375629)
$K_v7.2$	S352P	Helix A-B linker	c.1054T>C	Missense	Mild/BFNE	Grinton BE et al., 2015 (PMID 25982755)
$K_v7.2$	R353H	Helix A-B linker	c.1058G>A	Missense	Severe/EE	Milh M et al., 2015 (PMID 25959266)
$K_v7.2$	R353G	Helix A-B linker	c.1057C<G	Missense	Mild/BFNE	Grinton BE et al., 2015 (PMID 25982755); Zhou X et al., 2016 (PMID 28078031); Sands T et al., 2016 (PMID 27888506); Cavaretta JP et al., 2014 (PMID 25077630)
$K_v7.2$	L356V	Helix A-B linker	c.1066C>G	Missense	Severe/EE	Milh M et al., 2015 (PMID 25959266)
$K_v7.2$	S358F	Helix A-B linker	c.1073C>T	Missense	Mild/BFNE	Grinton BE et al., 2015 (PMID 25982755)
$K_v7.2$	T359K	Helix A-B linker	c.1076C>A	Missense	Mild/BFNE	Volkers L et al., 2009 (PMID 19559753)
$K_v7.2$	Y362C	Helix A-B linker	c.1085A>G	Missense	Mild/BFNE	Soldovieri MV et al., 2013 (PMID 24375629)
$K_v7.2$	P420M	Helix A-B linker	c.1259C>T	Missense	Severe/EE	Milh M et al., 2015 (PMID 25959266)
$K_v7.2$	V422I	Helix A-B linker	c.1264G>A	Missense	Severe/EE	Milh M et al., 2015 (PMID 25959266)
$K_v7.2$	E484D	Helix A-B linker	c.1452G>C	Missense	Severe/EE	Zhang Q et al., 2017 (PMID 27779742)
$K_v7.2$	A501P	Helix A-B linker	c.1501G>C	Missense	Severe/EE	Milh M et al., 2015 (PMID 25959266)
$K_v7.2$	E515D	Helix A-B linker	c.1545G>C	Missense	Mild/BFNE	Lee et al., 2016 (PMID 28038823); Lee IC et al., 2009 (PMID 19380078); Kato T et al., 2015 (PMID 25819767)
$K_v7.2$	R541G	Helix B	c.1621A>G	Missense	Severe/EE	Milh M et al., 2015 (PMID 25959266)
$K_v7.2$	M546V	Helix B	c.1636A>G	Missense	Severe/EE	Weckhuysen S et al., 2012 (PMID 22275249); Orhan et al., 2014 (PMID 24318194); Kim EC et al., 2018 (PMID 30008368)
$K_v7.2$	R547W	Helix B	c.1639C>T	Missense	Severe/EE	Zara F et al., 2013 (PMID 23360469)
$K_v7.2$	K552T	Helix B	c.1655A>C	Missense	Severe/EE	Weckhuysen S et al., 2013 (PMID 24107868); Pisano T et al., 2015 (PMID 25880994)
$K_v7.2$	R553W	Helix B	c.1657C>T	Missense	Mild/BFNE	Hortiguela M et al., 2017 (PMID 27535030)
$K_v7.2$	R553Q	Helix B	c.1685G>A	Missense	Mild/BFNE	Soldovieri MV et al., 2013 (PMID 24375629)
$K_v7.2$	R553L	Helix B	c.1658G>T	Missense	Severe/EE	Kato M et al., 2013 (PMID 23621294)
$K_v7.2$	K554N	Helix B	c.1662G>C /T	Missense	Uncertain severity	Steinlein OK et al., 2007 (PMID 17129708); Borgatti R et al., 2004 (PMID 15249611); Chung HJ et al.,2006 (PMID 16735477); Kim ECet al., 2018 (PMID 30008368)
$K_v7.2$	K556E	Helix B-C linker	c.1666A>G	Missense	Severe/EE	Weckhuysen S et al., 2013 (PMID 24107868); Pisano T et al., 2015 (PMID 25880994)
$K_v7.2$	R560W	Helix B-C linker	c.1678C>T	Missense	Severe/EE	Pisano T et al., 2015 (PMID 25880994); Weckhuysen S et al., 2012 (PMID 22275249); Orhan et al., 2014 (PMID 24318194); Kim EC et al., 2018 (PMID 30008368)
$K_v7.2$	P561S	Helix B-C linker	c.1681C>T	Missense	Severe/EE	Trump et al., 2016 (PMID 26993267)

$K_v7.2$	P561L	Helix B-C linker	c.1682C>T	Missense	Severe/EE	Kato M et al., 2013 (PMID 23621294)
$K_v7.2$	D563N	Helix B-C linker	c.1687G>A	Missense	Severe/EE	Weckhuysen S et al., 2013 (PMID 24107868)
$K_v7.2$	D563E	Helix B-C linker	c.1689C>G	Missense	Severe/EE	Kato M et al., 2013 (PMID 23621294)
$K_v7.2$	V567D	Helix B-C linker	c.1700T>A	Missense	Mild/BFNE	Sands T et al., 2016 (PMID 27888506)
$K_v7.2$	M578V	Helix C	c.1732A>G	Missense	Mild/BFNE	Grinton BE et al., 2015 (PMID 25982755)
$K_v7.2$	M578I	Helix C	c.1734G>C	Missense	Severe/EE	Numis AL et al., 2014 (PMID 24371303); Pisano T et al., 2015 (PMID 25880994)
$K_v7.2$	R581Q	Helix C	c.1742G>A	Missense	Severe/EE	Parrini et al., 2017 (PMID 27864847)
$K_v7.2$	R581G	Helix C	c.1741C>A	Missense	Severe/EE	Weckhuysen S et al., 2013 (PMID 24107868)
$K_v7.2$	R588S	Helix C	c.1764A>T	Missense	Mild/BFNE	Grinton BE et al., 2015 (PMID 25982755)
$K_v7.2$	I592M	Helix C-D linker	c.1776C>G	Missense	Mild/BFNE	Hahn A & Neubauer BA, 2009 (PMID 19464834); Neubauer BA et al., 2008 (PMID 18625963)
$K_v7.2$	R595W	Helix C-D linker	c.1783C>T	Missense	Mild/BFNE	Dyment DA et al., 2015 (PMID 25046240)
$K_v7.2$	L637R	Helix D	c.1910T>G	Missense	Mild/BFNE	Alberdi et al., 2015 (PMID 26359296); Grinton BE et al., 2015 (PMID 25982755)
$K_v7.2$	R871S	C-terminus	c.2613G>T	Missense	Severe/EE	Milh M et al., 2015 (PMID 25959266)
$K_v7.2$	No protein	NA	c.(?_-177)_690+?del	Submicroscopic Deletion	Mild/BFNE	Soldovieri MV et al., 2013 (PMID 24375629)
$K_v7.2$	No protein	NA	c.1-?c.993+?del	Submicroscopic Deletion	Mild/BFNE	Grinton BE et al., 2015 (PMID 25982755); Heron SE et al., 2007 (PMID 17675531)
$K_v7.2$	No protein	NA	c.474-940_c.1424+1582del	Submicroscopic Deletion	Mild/BFNE	Grinton BE et al., 2015 (PMID 25982755); Heron SE et al., 2007 (PMID 17675531)
$K_v7.2$	No protein	NA	c.565-682_c.1295+del	Submicroscopic Deletion	Mild/BFNE	Grinton BE et al., 2015 (PMID 25982755)
$K_v7.2$	No protein	NA	c.565-?_c.1478+?(2)	Submicroscopic Deletion	Mild/BFNE	Grinton BE et al., 2015 (PMID 25982755); Heron SE et al., 2007 (PMID 17675531)
$K_v7.2$	No protein	NA	c.1119-?_(*382)del	Submicroscopic Deletion	Mild/BFNE	Singh NA et al., 2003 (PMID 14534157); Singh NA et al., 1998 (PMID 9425895)
$K_v7.2$	No protein	NA	c.1479-768_c.1940+579del3018bp	Submicroscopic Deletion	Mild/BFNE	Grinton BE et al., 2015 (PMID 25982755); Heron SE et al., 2007 (PMID 17675531)
$K_v7.2$	No protein	NA	c.1764-?_(*455_?)del	Submicroscopic Deletion	Mild/BFNE	Soldovieri MV et al., 2013 (PMID 24375629)
$K_v7.2$	DEL ex12-17	NA	DEL ex12-17	Partial gene deletion	Mild/BFNE	Sands T et al., 2016 (PMID 27888506)
$K_v7.2$	DEL ex13-17	NA	DEL ex13-17	Partial gene deletion	Mild/BFNE	Sands T et al., 2016 (PMID 27888506)
$K_v7.2$	V99?	S1	c.296+1G>A; c.297-2A>G	Splice-site	Mild/BFNE	Steinlein OK et al., 2007 (PMID 17129708); Zara F et al., 2013 (PMID 23360469)
$K_v7.2$	L129?	S2	c.387+1G>T	Splice-site	Mild/BFNE	Singh NA et al., 2003 (PMID 14534157)
$K_v7.2$	E130?	S2	c.388-2_388delAGG	Splice-site	Mild/BFNE	Steinlein OK et al., 2007 (PMID 17129708)
$K_v7.2$	G310?	S6	c.928-1G>C	Splice-site	Mild/BFNE	Soldovieri MV et al., 2014 (PMID 24375629)
$K_v7.2$	S342?	Helix A	c.1024-2A>G	Splice-site	Uncertain severity	Milh M et al., 2015 (PMID 25959266)
$K_v7.2$	S373?	Helix A-B linker	c.1118+1G>A; c.1118+3A>G	Splice-site	Mild/BFNE	Claes LR et al., 2004 (PMID 15596769); Zara F et al., 2013 (PMID 23360469)
$K_v7.2$	R383?	Helix A-B linker	c.1148+2T>G	Splice-site	Uncertain severity	Lee WL et al., 2000 (PMID 10774989)
$K_v7.2$	R406?	Helix A-B linker	c.1217+2T>G	Splice-site	Mild/BFNE	Steinlein OK et al., 2007 (PMID 17129708)

$K_v7.2$	S416?	Helix A-B linker	c1247+1G>A	Splice-site	Mild/BFNE	Grinton BE et al., 2015 (PMID 25982755)
$K_v7.2$	S434?	Helix A-B linker	c.1302-1G>C	Splice-site	Mild/BFNE	Steinlein OK et al., 2007 (PMID 17129708)
$K_v7.2$	E509?	Helix A-B linker	c.1525+1G>A	Splice-site	Mild/BFNE	Steinlein OK et al., 2007 (PMID 17129708); Richards MC et al., 2004 (PMID 14985406)
$K_v7.2$	C544?	Helix B	c.1631+1G>A; c.1632-1G>T	Splice-site	Mild/BFNE	Steinlein OK et al., 2007 (PMID 17129708); Singh NA et al., 1998 (PMID 9425895); Grinton BE et al., (PMID 25982755)
$K_v7.2$	R588?	Helix C	1764-2A>G	Splice-site	Mild/BFNE	Steinlein OK et al., 2007 (PMID 17129708)
$K_v7.2$	Q629?	Helix D	c.1887+5G>A	Splice-site	Mild/BFNE	Zara F et al., 2013 (PMID 23360469)
$K_v7.2$	W157X	S2-S3 linker	c.471G>A	Nonsense	Uncertain severity	Milh M et al., 2013 (PMID 23692823)
$K_v7.2$	S195X	S3-S4 linker	c.584_593del_insA	Nonsense	Uncertain severity	Bassi MT et al., 2005 (PMID 16235065)
$K_v7.2$	G204X	S4	c.610C>T	Nonsense	Mild/BFNE	Grinton BE et al., 2015 (PMID 25982755)
$K_v7.2$	S247X	S5	c.740C>A	Nonsense	Mild/BFNE	Hunter J et al., 2006 (PMID 16916607)
$K_v7.2$	W269X	Pore	c.807G>A	Nonsense	Mild/BFNE	Singh NA et al., 2003 (PMID 14534157)
$K_v7.2$	Q323X	Pre-helix A	c.967C>T	Nonsense	Mild/BFNE	Chung HJ et al.,2006 (PMID 16735477); Singh NA et al., 2003 (PMID 14534157)
$K_v7.2$	S399X	Helix A-B linker	c.1195_1196delAG	Nonsense	Mild/BFNE	Soldovieri MV et al., 2014 (PMID 24375629)
$K_v7.2$	R448X	Helix A-B linker	c.1342C>T	Nonsense	Mild/BFNE	Zonana J et al., 1984 (PMID 6476007); Singh NA et al., 2003 (PMID 14534157); Richards MC et al., 2004 (PMID 14985406); Zara F et al., 2013 (PMID 23360469); Grinton BE et al., 2015 (PMID 25982755); Moulard B et al., 2001 (PMID 11690625); Yum MS et al., 2010 (PMID 20119593)
$K_v7.2$	K537X	Helix B	c.1609A>T	Nonsense	Mild/BFNE	Soldovieri MV et al., 2014 (PMID 24375629)
$K_v7.2$	R581X	Helix C	c.1741C>T	Nonsense	Mild/BFNE	Singh NA et al., 2003 (PMID 14534157); Grinton BE et al., 2015 (PMID 25982755)
$K_v7.2$	V22AfsX18	N-terminal	c.63_66delGGTG	Frameshift	Mild/BFNE	Goldberg-Stern H et al., 2009 (PMID 19818940); Grinton BE et al., 2015 (PMID 25982755)
$K_v7.2$	K69QfsX50	N-terminal	c.204_205insC	Frameshift	Mild/BFNE	Richards MC et al., 2004 (PMID 14985406); Grinton BE et al., 2015 (PMID 25982755)
$K_v7.2$	Q78RfsX54	N-terminal	c.232delC	Frameshift	Mild/BFNE	Claes LR et al., 2004 (PMID 15596769)
$K_v7.2$	S113HfsX6	S1-S2 linker	c.333_334delGT	Frameshift	Mild/BFNE	Soldovieri MV et al., 2014 (PMID 24375629)
$K_v7.2$	A196CfsX66	S4	c.585_586insT	Frameshift	Mild/BFNE	Moulard B et al., 2001 (PMID 11690625)
$K_v7.2$	R198KfsX63	S4	c.592_594delCGGinsA	Frameshift	Mild/BFNE	Zara F et al., 2013 (PMID 23360469)
$K_v7.2$	K283SfsX36	Pore	c.847_848insGT	Frameshift	Mild/BFNE	Singh NA et al., 1998 (PMID 9425895)
$K_v7.2$	S314VfsX16	Pre-helix A	c.939_940insG	Frameshift	Uncertain severity	Steinlein MV et al., 2007 (PMID 18698150)
$K_v7.2$	T376LfsX12	Helix A-B linker	c.1126delA	Frameshift	Mild/BFNE	Saadeldin IY et al., 2013 (PMID 23290024)
$K_v7.2$	K398EfsX1	Helix A-B linker	c.1192_1193delAA	Frameshift	Mild/BFNE	Pereira S et al., 2004 (PMID 15030501)
$K_v7.2$	P410fsX12	Helix A-B linker	c.1229?	Frameshift	Mild/BFNE	Volkers L et al., 2009 (PMID 19559753)
$K_v7.2$	S416RfsX57	Helix A-B linker	c.1246A>C	Frameshift	Mild/BFNE	Soldovieri MV et al., 2014 (PMID 24375629)
$K_v7.2$	Q429Rfs*5	Helix A-B linker	c.1284delG	Frameshift	Severe/EE	Zhang Q et al., 2017 (PMID 27779742)
$K_v7.2$	L473RfsX47	Helix A-B linker	c.1418_1419delTC	Frameshift	Mild/BFNE	Grinton BE et al., 2015 (PMID 25982755)
$K_v7.2$	Q494fsX	Helix A-B linker	c.1481?	Frameshift	Mild/BFNE	Lerche H et al., 2001 (PMID 11579435)
$K_v7.2$	C523WfsX1	Helix A-B linker	c.1569_1581del	Frameshift	Mild/BFNE	Singh NA et al., 1998 (PMID 9425895)

$K_v7.2$	Y562Cfs X4	Helix B-C linker	c.1684_1685duplication	Frameshift	Mild/BFNE	Grinton BE et al., 2015 (PMID 25982755); Biervert C et al., 1998); Chung HJ et al.,2006 (PMID 16735477)
$K_v7.2$	M619Rfs X13	Helix D	c.1856_1886del	Frameshift	Mild/BFNE	Grinton BE et al., 2015 (PMID 25982755); Schwake M et al., 2006 (PMID 16597729); Wehling C et al., 2007 (PMID 17382933)
$K_v7.2$	V630SfsX 12	Helix D	c.1888delG	Frameshift	Mild/BFNE	Sands T et al., 2016 (PMID 27888506)
$K_v7.2$	Y644Tfs X285	Helix D	c.1930delT	Frameshift	Mild/BFNE	Biervert C et al., 1998 (PMID 9430594); Schwake M et al., 2006 (PMID 16597729); Wehling C et al., 2007 (PMID 17382933); Steinlein MV et al., 2007 (PMID 18698150)
$K_v7.2$	T653Qfs X285	C-terminal	c.1957delG	Frameshift	Mild/BFNE	Singh NA et al., 2003 (PMID 14534157)
$K_v7.2$	S672TfsX 257	C-terminal	c.2015delG	Frameshift	Mild/BFNE	Tang B et al., 2004 (PMID 15178210)
$K_v7.2$	V710SfsX 219	C-terminal	c.2127delT	Frameshift	Mild/BFNE	Coppola G et al., 2003 (PMID 12847176); Zimprich F et al., 2006 (PMID 16966552); Chung HJ et al., 2006 (PMID 16735477); Soldovieri MV et al., 2007 (PMID 18698150)
$K_v7.2$	C774LfsX 90	C-terminal	c.2318_2319dupG	Frameshift	Uncertain severity	Milh M et al., 2013 (PMID 23692823)
$K_v7.2$	G866Afs X63	C-terminal	c.2597delG	Frameshift	Mild/BFNE	Lerche H et al., 2001 (PMID 11579435); Chung HJ et al., 2006 (PMID 16735477); Steinlein OK et al., 2007 (PMID 17129708); Su J et al., 2011 (PMID 21937445)
$K_v7.2$	R871Gfs X61	C-terminal	c.2599_2603duplication	Frameshift	Mild/BFNE	Soldovieri MV et al., 2014 (PMID 24375629)
$K_v7.2$	R871Gfs X60	C-terminal	c.2606_2610duplication	Frameshift	Mild/BFNE	Su J et al., 2011 (PMID 21937445); Singh NA et al., 2003 (PMID 14534157)

Missense and single amino acid mutations in $K_v7.2$ which were identified until 12/31/2017 were compiled and used in our statistical analyses. *Although M1V and M1T mutations in $K_v7.2$ were reported (RIKEE database: www.rikee.org), these start codon mutations were not included in the statistical analyses because such mutations most likely lead to the loss of primary start codon ATG for methionine. Epileptic Encephalopathy (EE); Benign Familial Neonatal Epilepsy (BFNE); NA (Not Applicable)

Supplementary Table S2. Identification of pathogenic epilepsy mutation clusters in $K_v7.2$ using the MHF algorithms on the full length $K_v7.2$ protein sequence or C-terminal tail only.

				**Pathogenic**			**Silent**			**Nonpathogenic**		
Protein	Domain	Location		Number of Mutations		*P*-Value Adj.	Number of Mutations		*P*-Value Adj.	Number of Mutations		*P*-Value Adj.
		Start	End	Obs. No.	Exp. No.		Obs. No.	Exp. No.		Obs. No.	Exp. No.	
$K_v7.2$ Full Length	S1	92	112	2	3.125	1	3	3.127	1	0	0.616	1
	S2	123	143	1	3.128	1	3	3.135	1	0	0.602	1
	S2-S3 Linker	144	166	4	3.424	1	3	3.451	1	0	0.650	1
	S3	167	187	2	3.119	1	1	3.126	1	0	0.611	1
	S4	196	218	18	3.403	0.0014*	3	3.402	1	0	0.662	1
	S4-S5 Linker	219	231	1	1.923	1	1	1.924	1	0	0.376	1
	S5	232	252	8	3.126	0.2002	3	3.117	1	0	0.592	1
	Pore	253	291	29	5.823	0.0014*	8	5.852	1	0	1.130	1
	S6	292	312	11	3.151	0.0098*	6	3.152	1	0	0.608	1
	Helix A	324	350	7	4.035	1	0	4.039	1	0	0.770	1
	Helix B	532	555	8	3.598	0.3962	3	3.552	1	0	0.692	1
	Helix C	568	589	5	3.259	1	5	3.281	1	1	0.624	1
	Helix D	619	651	1	4.928	1	3	4.920	1	0	0.942	1
	Ankyrin-G Binding	832	848	0	2.515	1	5	2.523	1	0	0.488	1
$K_v7.2$ C-tail only	Pre-Helix A	313	323	1	0.916	1	0	1.760	1	0	0.465	1
	Helix A	324	350	7	2.229	0.0531	0	4.322	1	0	1.157	1
	Helix A-B Linker	351	531	14	14.883	1	23	29.109	1	4	7.749	1
	Helix B	532	555	8	1.973	0.0063*	3	3.834	1	0	1.038	1
	Helix B-C Linker	556	567	7	0.998	0.0018*	2	1.912	1	2	0.521	0.8577
	Helix C	568	589	5	1.805	0.2898	5	3.562	1	1	0.938	1
	Helix C-D Linker	590	618	2	2.386	1	6	4.624	1	1	1.245	1
	Helix D	619	651	1	2.695	1	3	5.343	1	0	1.411	1
	Ankyrin-G Binding	832	848	0	1.382	1	5	2.724	1	0	0.727	1

$N_{mutations}$ Observed (Obs.), the true number of mutations observed within the protein domain of full length $K_v7.2$ (NP_742105.1) and only C-tail of $K_v7.2$ (NP_742105.1); $N_{mutations}$ Expected (Exp.), the sample mean of the number of mutations expected within the protein domain of $K_v7.2$ under the null hypothesis. If p value adjusted (Adj.) from Bonferroni's method for multiple comparison correction is < 0.05, the domain is statistically determined to contain a mutation cluster and marked with "*".

Supplementary Table S3. Identification of mutation clusters in K_v7.2 which are associated with epilepsy of varying clinical severity using the MHF algorithms.

Protein	Domain	Nonpathogenic			Mild/BFNE			Uncertain Severity			Severe/EE		
		Obs. No.	Exp. No.	*P*-Value Adj.	Obs. No.	Exp. No.	*P*-Value Adj.	Obs. No.	Exp. No.	*P*-Value Adj.	Obs. No.	Exp. No.	*P*-Value Adj.
K_v7.2	S1	0	0.598	1	2	1.030	1	0	0.124	1	0	1.954	1
	S2	0	0.584	1	0	1.042	1	0	0.117	1	1	1.950	1
	S2-S3 Linker	0	0.659	1	3	1.147	1	0	0.135	1	1	2.153	1
	S3	0	0.603	1	0	1.028	1	0	0.120	1	2	1.984	1
	S4	0	0.656	1	4	1.127	0.363	3	0.136	0.006*	11	2.182	0.003*
	S4-S5 Linker	0	0.381	1	1	0.626	1	0	0.076	1	0	1.222	1
	S5	0	0.584	1	2	1.031	1	0	0.120	1	6	1.972	0.200
	Pore	0	1.132	1	5	1.958	0.634	1	0.230	1	23	3.680	0.001*
	S6	0	0.616	1	3	1.028	1	0	0.119	1	8	1.985	0.008*
	Helix A	0	0.783	1	3	1.323	1	0	0.151	1	4	2.536	1
	Helix B	0	0.685	1	2	1.190	1	1	0.135	1	5	2.245	1
	Helix C	1	0.631	1	2	1.081	1	0	0.125	1	3	2.083	1
	Helix D	0	0.956	1	1	1.629	1	0	0.189	1	0	3.113	1
	Ankyrin-G Binding	0	0.491	1	0	0.836	1	0	0.095	1	0	1.611	1
K_v7.2 C-tail only	Pre-Helix A	0	0.477	1	0	0.369	1	0	0.021	1	1	0.509	1
	Helix A	0	1.172	1	3	0.920	0.573	0	0.047	1	4	1.250	0.3051
	Helix A-B Linker	4	7.734	1	8	6.120	1	0	0.323	1	6	8.410	1
	Helix B	0	1.044	1	2	0.816	1	1	0.041	0.373	5	1.113	0.046*
	Helix B-C Linker	2	0.513	0.838	1	0.415	1	0	0.022	1	6	0.564	9.0E-04*
	Helix C	1	0.942	1	2	0.740	1	0	0.041	1	3	1.029	0.760
	Helix C-D Linker	1	1.246	1	2	0.988	1	0	0.053	1	0	1.356	1
	Helix D	0	1.408	1	1	1.123	1	0	0.054	1	0	1.532	1
	Ankyrin-G Binding	0	0.733	1	0	0.573	1	0	0.030	1	0	0.800	1

$N_{mutations}$ Observed (Obs.), the true number of mutations observed within the protein domain of full-length K_v7.2 and K_v7.2 C-terminal tail only (NP_742105.1); $N_{mutations}$ Expected (Exp.), the sample mean of the number of mutations expected within the protein domain of full-length K_v7.2 and intracellular C-terminal tail of K_v7.2 under the null hypothesis. If p value adjusted (Adj.) from Bonferroni's method for multiple comparison correction is < 0.001, the domain is statistically determined to contain a mutation cluster and marked with "*".

Supplementary Table S4. Passive properties of the GFP-positive CHO hm1 cells transfected with wild type K_v7.2 or EE mutant K_v7.2 as well as the cells transfected with K_v7.3, wild type K_v7.2 and EE mutant K_v7.2 at a 2:1:1 ratio.

Transfection		*n*	V_m (mV)	E_{rev} (mV)	C_m (pF)
GFP + K_v7.2	WT	12	-35.5 ± 1.1	-38.8 ± 1.9	17.3 ± 0.6
	L203P	17	-26.8 ± 2.0*	-22.1 ± 1.2*	17.6 ± 0.3
	L268F	17	-42.1 ± 1.8*	-43.7 ± 2.2	17.8 ± 0.4
	K552T	13	-32.1 ± 2.1	-32.9 ± 2.0	16.6 ± 0.4
	R553L	13	-38.3 ± 0.7*	-36.6 ± 1.1	16.8 ± 1.5
	WT + diC8-PIP_2	11	-42.5 ± 1.8	-39.8 ± 1.3	18.0 ± 0.6
	L203P + diC8-PIP_2	14	-31.0 ± 2.6†	-24.1 ± 1.4†‡	17.1 ± 0.8
	L268F + diC8-PIP_2	13	-44.0 ± 2.1	-45.2 ± 1.9	17.6 ± 0.4
	K552T + diC8-PIP_2	11	-35.2 ± 2.2†	-36.5 ± 2.7	17.0 ± 0.8
	R553L + diC8-PIP_2	11	-37.6 ± 2.1†	-38.7 ± 1.6	16.8 ± 1.6
GFP + K_v7.3 + K_v7.2	WT	14	-46.6 ± 0.8	-50.2 ±1.2	18.7 ± 0.5
	L203P	15	-47.5 ± 1.0	-48.0 ± 1.0	18.6 ± 0.9
	L268F	18	-46.8 ± 0.8	-49.2 ± 1.3	18.2 ± 0.5
	K552T	15	-45.6 ± 1.1	-48.7 ± 1.8	16.9 ± 1.1
	R553L	15	-47.2 ± 0.7	-53.1 ± 2.6	18.9 ± 1.1
	WT + diC8-PIP_2	12	-48.3 ± 1.3	-49.8 ± 1.4	18.1 ± 1.2
	L203P + diC8-PIP_2	12	-44.8 ± 1.4	-44.5 ± 1.0	18.2 ± 0.8
	L268F + diC8-PIP_2	16	-47.9 ± 0.8	-50.1 ± 1.0	18.7 ± 1.2
	K552T + diC8-PIP_2	14	-42.1 ± 0.9†	-46.4 ± 1.4	16.6 ± 1.3
	R553L + diC8-PIP_2	15	-47.8 ± 1.1	-53.7 ± 2.2	18.9 ± 0.7

n, number; V_m, resting membrane potential; E_{rev} (mV), reversal potential; C_m, whole cell membrane capacitance. Ave ± SEM (GFP+EE variants: *$p<0.05$ for K_v7.2 WT vs. mutant; †$p<0.05$ for K_v7.2 WT + diC8-PIP_2 vs. mutant + diC8-PIP_2; ‡$p<0.05$ for K_v7.2 WT vs mutants + diC8-PIP_2; ^$p<0.05$ for the difference between - diC8-PIP_2 and + diC8-PIP_2 within the same transfection; GFP+K_v7.3+K_v7.2 WT+K_v7.2 mutant: *$p<0.05$ for K_v7.2 WT vs. mutant; †$p<0.05$ for K_v7.2 WT + diC8-PIP_2 vs. mutant + diC8-PIP_2; ‡$p<0.05$ for K_v7.2 WT vs mutants + diC8-PIP_2; ^$p<0.05$ for the difference between - diC8-PIP_2 and + diC8-PIP_2 within the same transfection). p-values are computed from post-hoc Tukey's test. E_{rev} corresponds to: Supplementary Fig. S1 (homomeric channel), Supplementary Fig. S5 (heteromeric channel).

Supplementary Table S5. Biophysical properties of homomeric $K_v7.2$ channels and heteromeric $K_v7.3$/ $K_v7.2$ channels in GFP-positive CHO hm1 cells.

Transfection		*n*	G/Gmax $V_{1/2}$ (mV)	G/Gmax *k* (mV/efold)	Leak-subtracted I at -20 mV (pA)	Leak-subtracted I at +20 mV (pA)
GFP + $K_v7.2$	WT	12	-26.8 ± 2.1	12.0 ± 1.1	88.5 ± 12.0	296.4 ± 16.6
	L203P	17	-3.6 ± 1.4*	9.3 ± 0.5	35.2 ± 4.5	482.4 ± 29.9*
	L268F	17	-30.8 ± 1.0	12.7 ± 0.6	110.0 ± 12.7	211.2 ± 24.5
	K552T	13	-32.5 ± 2.0	13.4 ± 0.7	37.0 ± 4.9	101.5 ± 16.9
	R553L	13	-31.6 ± 2.1	11.6 ± 1.0	118.5 ± 18.6	356.5 ± 58.3
	WT + diC8-PIP_2	11	-31.7 ± 1.5	9.2 ± 1.0	88.5 ± 12.0^	689.4 ± 46.9^
	L203P + diC8-PIP_2	14	-4.0 ± 2.1†‡	9.5 ± 0.7	29.1 ± 3.1†	413.2 ± 46.8†
	L268F + diC8-PIP_2	13	-35.0 ± 2.0	14.1 ± 0.8†	142.9 ± 31.5†	284.2 ± 72.3†
	K552T + diC8-PIP_2	11	-34.8 ± 2.3	17.3 ± 1.6†‡	41.8 ± 8.4†	107.8 ± 20.9†
	R553L + diC8-PIP_2	11	-34.6 ± 2.9	10.4 ± 1.0	137.4 ± 22.9†	400.2 ± 52.0†
GFP + $K_v7.3$ + $K_v7.2$	WT	14	-29.4 ± 0.9	11.3 ± 0.4	458.4 ± 28.8	1317.8 ± 50.3
	L203P	15	-31.3 ± 1.9	10.6 ± 0.5	573.1 ± 72.9	1569.1 ±166.1
	L268F	18	-34.3 ± 1.7	10.9 ± 0.3	250.9 ± 25.3*	628.1 ± 74.3*
	K552T	15	-28.6 ± 2.2	9.8 ± 0.9	207.2 ± 29.5*	531.2 ± 70.8*
	R553L	15	-33.9 ± 1.7	10.5 ± 0.4	488.2 ± 50.3	1252.5 ± 125.1
	WT + diC8-PIP_2	12	-33.1 ± 0.9	9.3 ± 0.3	712.5 ± 46.8*	1863.6 ± 70.9^
	L203P + diC8-PIP_2	12	-29.3 ± 2.0	10.2 ± 0.3	376.7 ± 43.9†	1411.6 ± 125.5
	L268F + diC8-PIP_2	16	-31.1 ± 1.1	10.5 ± 0.4	396.2 ± 46.0†	908.8 ± 108.6†
	K552T + diC8-PIP_2	14	-29.9 ± 1.6	10.9 ± 0.8	233.9 ± 28.8†‡	542.3 ± 67.9†
	R553L + diC8-PIP_2	15	-31.5 ± 1.6	10.8 ± 0.6	473.6 ± 52.3†	1269.1 ± 150.6†

n, number; $V_{1/2}$, half-activation potential; *k*, the slope factor; I, current (pA). All values are calculated from leak subtracted current. $V_{1/2}$ and *k* are calculated from normalized conductance G/Gmax. Ave ± SEM (GFP+EE variants: *p<0.05 for $K_v7.2$ WT vs. mutant; †p<0.05 for $K_v7.2$ WT + diC8-PIP_2 vs. mutant + diC8-PIP_2; ‡p<0.05 for $K_v7.2$ WT vs mutants + diC8-PIP_2; ^p<0.05 for the difference between - diC8-PIP_2 and + diC8-PIP_2 within the same transfection; GFP+$K_v7.3$+$K_v7.2$ WT+$K_v7.2$ mutant: *p<0.05 for $K_v7.2$ WT vs. mutant; †p<0.05 for $K_v7.2$ WT + diC8-PIP_2 vs. mutant + diC8-PIP_2; ‡p<0.05 for $K_v7.2$ WT vs mutants + diC8-PIP_2; ^p<0.05 for the difference between - diC8-PIP_2 and + diC8-PIP_2 within the same transfection). p-values are computed from post-hoc Tukey's test. $V_{1/2}$ and *k* correspond to: Supplementary Fig. S1-2 (homomeric channel) and Supplementary Fig. S5-6 (heteromeric channel).

Supplementary Table S6. Passive properties of the CHO hm1 cells transfected GFP, K_v7.2 wild type or EE mutant, and the CHO hm1 cells transfected GFP, K_v7.2 wild type or EE mutant, and PIP5K.

	Without PIP5K				With PIP5K			
Transfection (GFP +)	*n*	V_m (mV)	C_m (pF)	E_{rev} (mV)	*n*	V_m (mV)	C_m (pF)	E_{rev} (mV)
K_v7.2 WT	6	-37.8 ± 1.1	16.9 ± 0.6	-44.9 ± 1.6	6	-49.6 ± 0.5^^^	16.6 ± 0.3	-60.5 ± 1.3^^
K_v7.2 L203P	5	-37.1 ± 1.4	17.2 ± 0.8	-33.8 ± 0.4*	8	-35.7 ± 1.4†††	17.7 ± 0.7	-37.3 ± 0.9†††
K_v7.2 L268F	11	-42.2 ± 1.3	17.0 ± 0.4	-46.6 ± 2.4	10	-43.2 ± 1.8†	16.7 ± 0.6	-47.5 ± 3.3††
K_v7.2 K552T	7	-35.0 ± 2.1	17.2 ± 0.6	-38.4 ± 2.7	7	-37.0 ± 1.5†††	15.6 ± 0.7	-40.4 ± 2.3†††
K_v7.2 R553L	10	-37.8 ± 0.9	16.9 ± 0.4	-41.9 ± 1.1	9	-40.1 ± 0.8†††	16.5 ± 0.7	-49.5 ± 1.5†

n, number; V_m, resting membrane potential; E_{rev} (mV), reversal potential; C_m, whole cell membrane capacitance. Ave ± SEM (GFP+EE variants: *$p<0.05$ for K_v7.2 WT vs. mutant; †$p<0.05$ for K_v7.2 WT + PIP5K vs. mutant + PIP5K; ^$p<0.05$ for the difference between – PIP5K and + PIP5K within the same transfection). p-values are computed from post-hoc Tukey's test. E_{rev} was calculated from the raw current traces for Supplementary Fig. S3 (which show leak subtracted current traces and data analysis).

Supplementary Table S7. The list of EE mutations reported on the modeled tetrameric human $K_v7.2$ structure in Figure 8.

Protein	Mutation	Location	cDNA change	Mutation type	Primary Disease	Reference
$K_v7.2$	T114I	S1-S2 linker	c.341C>T	Missense	Severe/EE	Saitsu S et al., 2012 (PMID 22926866)
$K_v7.2$	E130K	S2	c.388G>A	Missense	Severe/EE	Hortiguela M et al., 2017 (PMID 27535030)
$K_v7.2$	R144Q	S2-S3 linker	c.431G>A	Missense	Severe/EE	Miceli F et al., 2015 (PMID 25740509); Allen AS et al., 2013 (PMID 23934111)
$K_v7.2$	V175L	S3	c.523G>T; c.523G>C	Missense	Severe/EE	Milh M et al., 2013 (PMID 23692823); Devaux J et al., 2016 (PMID 27030113); Samanta D et al, 2015 (PMID 25092550)
$K_v7.2$	S187Y	S3	c.560C>A	Missense	Severe/EE	Gokben S et al., 2017 (PMID 27734276)
$K_v7.2$	G189V	S3-S4 linker	c.566G>T	Missense	Severe/EE	Milh et al., 2013 (PMID 236928230); Milh M et al., 2015 (PMID 25959266)
$K_v7.2$	N190S	S3-S4 linker	c.569A>G	Missense	Severe/EE	Millichap et al., 2016 (PMID 27602407)
$K_v7.2$	S195P	S3-S4 linker	c.583T>C	Missense	Severe/EE	Weckhuysen S et al., 2013 (PMID 24107868)
$K_v7.2$	R198Q	S4	c.593G>A	Missense	Severe/EE	Millichap et al., 2017 (PMID 27861786)
$K_v7.2$	R201C	S4	c.601C>T	Missense	Severe/EE	Trump et al., 2016 (PMID 26993267); Millichap et al., 2016 (PMID 27602407); Weckhuysen S et al., 2013 (PMID 24107868); Hortiguela et al., 2017 (PMID 27535030); Miceli F et al., 2015 (PMID 25740509)
$K_v7.2$	R201H	S4	c.602G>A	Missense	Severe/EE	Carvill GL et al., 2013 (PMID 23708187); Miceli F et al., 2015 (PMID 25740509); Pisano T et al., 2015 (PMID 25880994)
$K_v7.2$	L203P	S4	c.608T>C	Missense	Severe/EE	Milh M et al., 2013 (PMID 23692823)
$K_v7.2$	I205V	S4	c.613A>G	Missense	Severe/EE	Pisano T et al., 2015 (PMID 25880994); Niday Z et al., 2017 (PMID 28100740); Weckhuysen S et al., 2012 (PMID 22275249); Orhan G et al., 2014 (PMID 24318194)
$K_v7.2$	R210H	S4	c.629G>A	Missense	Severe/EE	Reid et al., 2016 (PMID 26446091); Numis AL et al., 2014 (PMID 24371303); Pisano T et al., 2015 (PMID 25880994); Weckhuysen et al., 2013 (PMID 24107868)
$K_v7.2$	R210C	S4	c.628C>T	Missense	Severe/EE	Mercimek-Mahmutoglu S et al., 2015 (PMID 25818041)
$K_v7.2$	R213W	S4	c.637C>T	Missense	Severe/EE	Zhang Q et al., 2017 (PMID 27779742); Trump et al., 2016 (PMID 26993267); Milh M et al., 2015 (PMID 25959266)
$K_v7.2$	R213Q	S4	c.638G>A	Missense	Severe/EE	Trump et al., 2016 (PMID 26993267); Grinton BE et al., 2015 (PMID 25982755); Pisano T et al., 2015 (PMID 25880994); Weckhuysen S et al., 2012 (PMID 22275249); Orhan G et al., 2014 (PMID 24318194)
$K_v7.2$	G215R	S4	c.643G>A	Missense	Severe/EE	Meurs-van der Schoor et al., 2014 (PMID 25566516)
$K_v7.2$	T217N	S4	c.650C>A	Missense	Severe/EE	Kato M et al., 2013 (PMID 23621294); Surti TS et al., 2005 (PMID 16319223)
$K_v7.2$	T234P	S5	c.700A>C	Missense	Severe/EE	Mercimek-Mahmutoglu S et al., 2015 (PMID 25818041)
$K_v7.2$	Y237F	S5	c.710A>T	Missense	Severe/EE	Zhang Q et al., 2017 (PMID 27779742)
$K_v7.2$	G239R	S5	c.715G>C	Missense	Severe/EE	Milh M et al., 2015 (PMID 25959266); Milh M et al., 2013 (PMID 23692823)
$K_v7.2$	A246P	S5	c.736G>C	Missense	Severe/EE	Zhang Q et al., 2017 (PMID 27779742)
$K_v7.2$	S247W	S5	c.740C>G	Missense	Severe/EE	Steinlein OK et al., 2007 (PMID 17129708); Kato M et al., 2013 (PMID 23621294); Dedek et al., 2003 (PMID 11572947)
$K_v7.2$	V250L	S5	c.748G>T	Missense	Severe/EE	Zhang Q et al., 2017 (PMID 27779742)
$K_v7.2$	A253T	Pore	c.757G>A	Missense	Severe/EE	Milh M et al., 2015 (PMID 25959266);
$K_v7.2$	G256W	Pore	c.766G>T	Missense	Severe/EE	Millichap et al.,2016 (PMID 27602407)

K_v7.2	A265T	Pore	c.793G>A	Missense	Severe/EE	Zhang Q et al., 2017 (PMID 27779742); Millichap et al.,2016 (PMID 27602407); Hortiguela M et al., 2017 (PMID 27535030); Milh M et al., 2013 (PMID 23692823); Weckhuysen S et al., 2013 (PMID 24107868)
K_v7.2	A265V	Pore	c.794C>T	Missense	Severe/EE	Saitsu S et al., 2012 (PMID 22926866); Milh M et al., 2015 (PMID 25959266)
K_v7.2	A265P	Pore	c.793G>C	Missense	Severe/EE	Weckhuysen S et al., 2012 (PMID 22275249); Orhan et al., 2014 (PMID 24318194)
K_v7.2	D266E	Pore	c.798T>A	Missense	Severe/EE	Parrini et al., 2017 (PMID 27864847)
K_v7.2	L268P	Pore	c.803T>C	Missense	Severe/EE	Hortiguela M et al., 2017 (PMID 27535030)
K_v7.2	L268F	Pore	c.802C>T	Missense	Severe/EE	Pisano T et al., 2015 (PMID 25880994)
K_v7.2	W269L	Pore	NR	Missense	Severe/EE	Millichap et al.,2016 (PMID 27602407)
K_v7.2	T274M	Pore	c.821C>T	Missense	Severe/EE	Zhang Q et al., 2017 (PMID 27779742); Millichap et al., 2016 (PMID 27602407); Hortiguela M et al., 2017 (PMID 27535030); Milh M et al., 2015 (PMID 25959266); Milh M et al., 2013 (PMID 23692823); Weckhuysen S et al., 2013 (PMID 24107868); Orhan et al., 2014 (PMID 24318194)
K_v7.2	T276I	Pore	c.827C>T	Missense	Severe/EE	Martin HC et al., 2014 (PMID 24463883)
K_v7.2	G279C	Pore	c.835G>T	Missense	Severe/EE	Milh M et al., 2015 (PMID 25959266)
K_v7.2	Y280H	Pore	c.838T>C	Missense	Severe/EE	Zhang Q et al., 2017 (PMID 27779742)
K_v7.2	G281W	Pore	c.841G>T	Missense	Severe/EE	Pisano T et al., 2015 (PMID 25880994)
K_v7.2	G281R	Pore	c.841G>A	Missense	Severe/EE	Weckhuysen S et al., 2013 (PMID 24107868)
K_v7.2	Y284H	Pore	c.850T>C	Missense	Severe/EE	Zhang Q et al., 2017 (PMID 27779742)
K_v7.2	Y284D	Pore	c.850T>G	Missense	Severe/EE	Hortiguela M et al., 2017 (PMID 27535030)
K_v7.2	P285H	Pore	c.854C>A	Missense	Severe/EE	Kato M et al., 2013 (PMID 23621294)
K_v7.2	T287N	Pore	c.860C>A	Missense	Severe/EE	Milh M et al., 2015 (PMID 25959266); Milh M et al., 2013 (PMID 23692823)
K_v7.2	G290S	Pore	c.868G>A	Missense	Severe/EE	Milh M et al., 2015 (PMID 25959266); Milh M et al., 2013 (PMID 23692823); Zhang Q et al., 2017 (PMID 27779742)
K_v7.2	G290D	Pore	c.869G>A	Missense	Severe/EE	Orhan et al., 2014 (PMID 24318194); Weckhuysen S et al., 2012 (PMID 22275249)
K_v7.2	R291S	Pore	c.873G>T	Missense	Severe/EE	Zhang Q et al., 2017 (PMID 27779742); Parrini et al., 2017 (PMID 27864847)
K_v7.2	R291G	Pore	c.871A>G	Missense	Severe/EE	Zhang Q et al., 2017 (PMID 27779742)
K_v7.2	A294V	S6	c.881C>T	Missense	Severe/EE	Parrini et al., 2017 (PMID 27864847); Millichap et al.,2016 (PMID 27602407); Hortiguela M et al., 2017 (PMID 27535030); Milh M et al., 2015 (PMID 25959266); Milh M et al., 2013 (PMID 23692823); Pisano T et al., 2015 (PMID 25880994); Abidi et al., 2015 (PMID 26007637)
K_v7.2	T296P	S6	c.886A>C	Missense	Severe/EE	Milh M et al., 2013 (PMID 23692823)
K_v7.2	G301S	S6	c.901G>A	Missense	Severe/EE	Parrini et al., 2017 (PMID 27864847); Milh M et al., 2015 (PMID 25959266)
K_v7.2	F304S	S6	c.911T>C	Missense	Severe/EE	Milh M et al., 2015 (PMID 25959266); Milh M et al., 2013 (PMID 23692823)
K_v7.2	F305del	S6	c.913_915delTTC	Single a.a. deletion	Severe/EE	Štěrbová K et. al., 2018 (PMID 29720203)
K_v7.2	F305L	S6	c.913T>C; c.915C>A	Missense	Severe/EE	Zhang Q et al., 2017 (PMID 27779742); Weckhuysen S et al., 2013 (PMID 24107868)
K_v7.2	A306V	S6	c.917C>T	Missense	Severe/EE	Hortiguela M et al., 2017 (PMID 27535030); Milh M et al., 2015 (PMID 25959266)
K_v7.2	A309V	S6	c.926C>T	Missense	Severe/EE	Milh M et al., 2015 (PMID 25959266); Milh M et al., 2013 (PMID 23692823)
K_v7.2	G315R	pre-helix A	c.943G>C	Missense	Severe/EE	Hortiguela M et al., 2017 (PMID 27535030); Weckhuysen S et al., 2013 (PMID 24107868)
K_v7.2	R325G	Helix A	c.973A>G	Missense	Severe/EE	Pisano T et al., 2015 (PMID 25880994); Soldovieri et al., 2016 (PMID 27905566); Weckhuysen S et al., 2013 (PMID 24107868)
K_v7.2	R333W	Helix A	c.997C>T	Missense	Severe/EE	Steinlein OK et al., 2007 (PMID 17129708); Schmitt et al., 2005 (PMID 16039833); Milh M et al., 2013 (PMID 23692823); Kim EC et al., 2018 (PMID 30008368)
K_v7.2	A337T	Helix A	NR	Missense	Severe/EE	Millichap et al.,2016 (PMID 27602407)

K_v7.2	A337G	Helix A	c.1010C>G	Missense	Severe/EE	Saitsu S et al., 2012 (PMID 22926866)
K_v7.2	R353H	Helix A-B linker	c.1058G>A	Missense	Severe/EE	Milh M et al., 2015 (PMID 25959266)
K_v7.2	L356V	Helix A-B linker	c.1066C>G	Missense	Severe/EE	Milh M et al., 2015 (PMID 25959266)
K_v7.2	P420M	Helix A-B linker	c.1259C>T	Missense	Severe/EE	Milh M et al., 2015 (PMID 25959266)
K_v7.2	V422I	Helix A-B linker	c.1264G>A	Missense	Severe/EE	Milh M et al., 2015 (PMID 25959266)
K_v7.2	E484D	Helix A-B linker	c.1452G>C	Missense	Severe/EE	Zhang Q et al., 2017 (PMID 27779742)
K_v7.2	A501P	Helix A-B linker	c.1501G>C	Missense	Severe/EE	Milh M et al., 2015 (PMID 25959266)
K_v7.2	R541G	Helix B	c.1621A>G	Missense	Severe/EE	Milh M et al., 2015 (PMID 25959266)
K_v7.2	M546V	Helix B	c.1636A>G	Missense	Severe/EE	Weckhuysen S et al., 2012 (PMID 22275249); Orhan et al., 2014 (PMID 24318194); Kim EC et al., 2018 (PMID 30008368)
K_v7.2	R547W	Helix B	c.1639C>T	Missense	Severe/EE	Zara F et al., 2013 (PMID 23360469)
K_v7.2	K552T	Helix B	c.1655A>C	Missense	Severe/EE	Weckhuysen S et al., 2013 (PMID 24107868); Pisano T et al., 2015 (PMID 25880994)
K_v7.2	R553L	Helix B	c.1658G>T	Missense	Severe/EE	Kato M et al., 2013 (PMID 23621294)
K_v7.2	K556E	Helix B-C linker	c.1666A>G	Missense	Severe/EE	Weckhuysen S et al., 2013 (PMID 24107868); Pisano T et al., 2015 (PMID 25880994)
K_v7.2	R560W	Helix B-C linker	c.1678C>T	Missense	Severe/EE	Pisano T et al., 2015 (PMID 25880994); Weckhuysen S et al., 2012 (PMID 22275249); Orhan et al., 2014 (PMID 24318194); Kim EC et al., 2018 (PMID 30008368)
K_v7.2	P561S	Helix B-C linker	c.1681C>T	Missense	Severe/EE	Trump et al., 2016 (PMID 26993267)
K_v7.2	P561L	Helix B-C linker	c.1682C>T	Missense	Severe/EE	Kato M et al., 2013 (PMID 23621294)
K_v7.2	D563N	Helix B-C linker	c.1687G>A	Missense	Severe/EE	Weckhuysen S et al., 2013 (PMID 24107868)
K_v7.2	D563E	Helix B-C linker	c.1689C>G	Missense	Severe/EE	Kato M et al., 2013 (PMID 23621294)
K_v7.2	M578I	Helix C	c.1734G>C	Missense	Severe/EE	Numis AL et al., 2014 (PMID 24371303); Pisano T et al., 2015 (PMID 25880994)
K_v7.2	R581Q	Helix C	c.1742G>A	Missense	Severe/EE	Parrini et al., 2017 (PMID 27864847)
K_v7.2	R581G	Helix C	c.1741C>A	Missense	Severe/EE	Weckhuysen S et al., 2013 (PMID 24107868)
K_v7.2	R871S	C-terminus	c.2613G>T	Missense	Severe/EE	Milh M et al., 2015 (PMID 25959266)
